# Almost Global Existence for Some Hamiltonian PDEs with Small Cauchy Data on General Tori

**DOI:** 10.1007/s00220-023-04899-z

**Published:** 2024-01-25

**Authors:** D. Bambusi, R. Feola, R. Montalto

**Affiliations:** 1 Dipartimento di Matematica, Università degli Studi di Milano, Via Saldini 50, 20133 Milan, Italy; 2 Dipartimento di Matematica e Fisica, Università degli Studi RomaTre, Largo San Leonardo Murialdo 1, 00144 Rome, Italy

## Abstract

In this paper we prove a result of almost global existence for some abstract nonlinear PDEs on flat tori and apply it to some concrete equations, namely a nonlinear Schrödinger equation with a convolution potential, a beam equation and a quantum hydrodinamical equation. We also apply it to the stability of plane waves in NLS. The main point is that the abstract result is based on a nonresonance condition much weaker than the usual ones, which rely on the celebrated Bourgain’s Lemma which provides a partition of the “resonant sites” of the Laplace operator on irrational tori.

## Introduction

The problem of studying long time behaviour of solutions of Hamiltonian non linear PDEs on compact manifolds is fundamental and widely studied. In this paper we focus on the so called problem of “almost global existence”, namely the problem of provingthat solutions corresponding to smooth and small initial data remain smooth and small for times of order $\epsilon ^{-r}$ with arbitrary *r*; here $\epsilon$ is the norm of the initial datum.

We recall that there exist quite satisfactory results for semilinear equations in one space dimension [1, 5, 19], which have also been extended to some semilinear PDEs with unbounded perturbations [47] and to some quasilinear wave equations [24], gravity capillary water waves [12] (see also [13]), capillary water waves [42], quasi-linear Schrödinger [32] and pure gravity water waves [14] still in dimension one. We also mention that for semilinear PDEs there are some results about sub-exponentially long stability time, see for instance [16, 17, 22, 30, 34].

On the other hand for the case of higher dimensional manifolds only particular examples are known [4, 11, 23, 26–28, 33] and for PDEs in higher space dimension with unbounded perturbations only partial results have been obtained [31, 35, 43]. A slightly different point of view is the one developed in [46] in which the authors give some upper bounds on the possible energy transfer to high modes, for initial data Fourier supported in a box for the cubic NLS on the irrational square torus in dimension two.

To discuss the main difficulty met in order to obtain almost global existence in more than one space dimension, we recall that all the known results deal with perturbations of linear systems whose eigenvalues are of the form $\pm i\omega _j$ with $\omega _j$ real numbers playing the role of frequencies. Here *j* belongs to some countable set of indexes, say $\mathbb {Z}^{d}$, $d\ge 1$ (for instance).

The main point is that, in all known results, the frequencies are assumed to verify a certain non-resonance condition. More precisely, for some fixed $\gamma ,\tau >0$, one typically requires

1.1 $$\begin{aligned} \left| \sum _{i=1}^{\ell }\omega _{j_i}-\sum _{k=\ell +1}^{r}\omega _{j_k}\right| \ge (\max _{3}\left\{ |j_1|,...,|j_r|\right\} )^{-\tau } \gamma , \end{aligned}$$

*except* in the case

1.2 $$\begin{aligned} r\;\;\textrm{even},\;\; \ell =\frac{r}{2}\;\;\;\mathrm{and\,(up\,to\,permutations)}\;\;\; j_i= j_{i+\tfrac{r}{2}}, \end{aligned}$$

where $\max _{3}\left\{ |j_1|,...,|j_r|\right\}$ denotes the third largest number among $|j_1|,...,|j_r|$. Condition (1.1) is a kind of second Melnikov condition since it requires to control linear combinations involving two frequencies with index arbitrarily large. Monomials in the vector field supported on indexes satisfying (1.2) are called *resonant monomials*, which are the ones that cannot be canceled out through a Birkhoff normal form procedure. We first remark that conditions (1.1)–(1.2) are quite strong, and, in particular, (1.2) implies that the only resonant monomials are *action preserving* in the sense that $|u_{j}|^{2}$ are constants of motion. Secondly one can easily convince that there are plenty of situation in which the conditions above are violated. Just as an example, even in dimension $d=1$ (in the case $j\in {\mathbb {Z}}$), and assuming $\omega _{j}$ even in *j* one can only hope to impose (1.1) except in the case

1.3 $$\begin{aligned} r\;\;\textrm{even},\;\; \ell =\frac{r}{2}\;\;\;\mathrm{and\,(up\,to\,permutations)}\;\;\; |j_i|= |j_{i+\tfrac{r}{2}}|, \end{aligned}$$

which is weaker than (1.2). Indeed it is no more true that the “actions” $|u_{j}|^{2}$ are preserved. On the contrary one can only infer that the so called *super-actions* are preserved by the motion, i.e. quantities of the form (in the case $d=1$)

$$\begin{aligned} |u_{j}|^{2}+|u_{-j}|^{2}. \end{aligned}$$

This suggests that the situation in which the linear system has *multiple* eigenvalues is more delicate. We mention, for instance, [29] where the authors deal with the multiplicity of the eigenvalues of the Laplacian on ${\mathbb {T}}^{d}$, $d>1$, by introducing the *super-actions*

$$\begin{aligned} J_{n}:=\sum _{k\in {\mathbb {Z}}^{d}, |k|^2=n}|u_{k}|^{2}. \end{aligned}$$

We finally remark that, apart from the possible multiplicity of eigenvalues, to have “good” lower bounds as in (1.1) is fundamental, in classical approaches, to prove the well-posedness of the Birkhoff map. It is also well known that to prove such lower bounds one needs to have *good separation properties* of the linear eigenvalues. For instance one can think of the Laplacian on ${\mathbb {T}}^1=\mathbb {S}^1$ where differences between eigenvalues *grows* at infinity since

$$\begin{aligned} ||j|^{2}-|k|^{2}|\ge |j|+|k|, \;\;\; \forall \, j,k\in {\mathbb {Z}}.\; |j|\ne |k|. \end{aligned}$$

This property holds, in some special cases also in high dimensions. For example it holds in the case of the Laplace-Beltrami operator on $\mathbb {S}^{d}$ and more in general holds for compact manifolds that are homogeneous with respect to a compact Lie Group of rank 1. These are special situations in high dimension in which it is still possible to prove bounds like (1.1), so essentially the problem of Birkhoff normal form can be treated as in the one dimensional case. These are the cases treated in [5, 6, 30]. Nevertheless, in general high dimensional settings differences of eigenvalues accumulate to zero (for example in the case of $\Delta$ on straight, irrational tori) and the Diophantine condition (1.1) is typically violated. We refer to [7] where properties of the Laplacian on general tori are discussed. In these more resonant cases it is anyway still possible to prove much *weaker Diophantine conditions* of the form

1.4 $$\begin{aligned} \left| \sum _{i=1}^{\ell }\omega _{j_i}-\sum _{k=\ell +1}^{r}\omega _{j_k}\right| \ge (\max \left\{ |j_1|,...,|j_r|\right\} )^{-\tau }\gamma \end{aligned}$$

for all possible choices of indexes $$j_1,...,j_r $$, except the case (1.3). *This is a condition typically fulfilled in any space dimension*. The crucial point is that condition (1.4) allows the small divisors to accumulate to zero *very* fast (as the largest index among $$|j_1|,\ldots , |j_{r}|$$ goes to infinity), and this could in principle create a *loss of derivatives* in the construction of the map used to put the system in Birkhoff normal form. We refer for instance to [11, 35, 43] (and reference therein) and where this problem is dealt with to prove partial long time stability results, by imposing (1.4) for small *r* (say $$r=3,4$$). By partial results we mean that, in the latter papers, the time scales of stability are of order at most $$\epsilon ^{-q}$$ with a strong limitation on $$q\le 4$$, and they left open the case *q* large.

In the present paper our aim is to develop a novel and self-contained framework in order to prove almost global existence (see Theorem 2.10 where *any* *r* are considered) for some Hamiltonian PDEs in which the linear frequencies are assumed to fulfil the weak condition (1.4).

The key point is that we also require the frequencies $\omega _j$ and the indexes *j* to fulfill a structural property ensured by a Lemma by Bourgain on the “localization of resonant sites” in ${\mathbb {T}}^d$. This allows to prove a theorem ensuring that the Hamiltonian of the PDE can be put in a suitable block-normal form which can be used to control the growth of Sobolev norms. For more details we refer the reader to the last paragraph of this introduction.

We emphasize that one of the points of interest of our paper is that it shows the impact of results of the kind of [15, 20, 25] dealing with linear time dependent systems on nonlinear systems, thus, in view of the generalizations [7–9], it opens the way to the possibility of proving almost global existence in more general systems, e.g. on some manifolds with integrable geodesic flow.

In the present paper, after proving the abstract result, we apply it to a few concrete equations for which almost global existence was out of reach with previous methods. Precisely we prove almost global existence of small amplitude solutions (1) for nonlinear Schrödinger equations with convolution potential, (2) for nonlinear beam equations and (3) for a quantum hydrodinamical model (QHD). We also prove Sobolev stability of plane waves for the Schrödinger equation (following [29]). We emphasize that these results were known only for the exceptional case of the *square torus*. We remark that our main theorem extends some partial results on the models listed above, we refer for instance to [33] for the QHD system (case (3) ) and [11] for the Beam equation (case (2)). For irrational tori the only result (as far as we know) ensuring at least a quadratic lifespan of nonlinear Schrödinger equations with unbounded, quadratic nonlinearities has been proved in [35]. The present paper, at least for semilinear nonlinearity, provides a method to prove polinomially long time stability for NLS on irrational tori.

To present in a more precise way the result, we recall that an arbitrary torus can be easily identified with the standard torus endowed by a flat metric. This is the point of view we will take. For the Schrödinger equation we show that, without any restrictions on the metric of the torus, one has that if the potential belongs to a set of full measure then one has almost global existence. For the case of the beam equation, we use the metric in order to tune the frequencies and to fulfill the nonresonance condition, thus we prove that if the metric of the torus is chosen in a set of full measure then almost global existence holds. Examples of tori fulfilling our property are rectangular tori with diophantine sides, but also more general tori are allowed.

The result for the QHD model is very similar to that of the beam equation: if the metric is chosen in a set of full measure, then almost global existence holds. Also the result of Sobolev stability of plane waves in the Schrödinger equation is of the same kind: if the metric belongs to a set of full measure, one has stability of the plane waves over times longer than any inverse power of $\epsilon$.

We also recall the result [10] in which the authors consider a nonlinear wave equation on ${\mathbb {T}}^d$ and prove that if the initial datum is small enough in some Sobolev norm then the solution remains small in a weaker Sobolev norm for times of order $\epsilon ^{-r}$ with arbitrary *r*. The main difference is that this result involves a loss of smoothness of the solution which is not present in our result; however, we emphasize that at present our method does no apply to the wave equation since no generalizations of Bourgain’s Lemma to systems of first order are known.

Finally we remark that our point of view is to show that solutions starting from a ball of radius $\epsilon$ do not reach the boundary of a ball of radius $2\epsilon$ for very long time. Proving this implies both the *existence* and the *stability* of the solution over a large time scale $O(\epsilon ^{-r})$. A different point of view is to give upper bounds on the possible growth of the Sobolev norm in terms of the time *t*. This problem, as already remarked, has been tackled widely for linear equations. However we mention [18, 44, 45] and the recent result [41] dealing with nonlinear equations. A dual point of view is to study possible *instability* of solutions, namely to show that even solutions evolving from *small* initial data could show a large growth of the Sobolev norm by waiting for sufficiently long time. Without trying to be exhaustive we quote [21, 36–40].

**Ideas of the proof of the abstract result.** Our aim is to study the dynamics of a Hamiltonian system whose corresponding Hamiltonian has an elliptic fixed point at the origin. Passing to the Fourier side and in appropriate complex coordinates $u_{j}$ we assume that the Hamiltonian has the form

$$\begin{aligned} H(u)=H_0+P(u), \qquad u=(u_{j})_{j\in {\mathbb {Z}}^{d}},\quad H_0:=\sum _{j\in {\mathbb {Z}}^{d}}\omega _{j}|u_{j}|^{2}, \end{aligned}$$

where $\omega _{j}$ are the linear frequencies of oscillations, the unknown *u* belongs to some scale of separable Hilbert spaces (we will work actually on scales of Sobolev spaces) and the perturbation $P=O(u^q)$ is a regular enough (say $C^{\infty }$) function having a zero at the origin of order at least $q\ge 3$. We also assume that *H* conserves the momentum. The precise assumptions on *H* are given in Sect. 2.2. By classical theory one expects that the homogeneous terms of high degree (at least *q* in this example) give a small contribution to the dynamics of the linear Hamiltonian. In other words, for *u* belonging to a small ball around the origin of order $\epsilon$ one expects a bound like $\epsilon ^{q-1}$ for the vector field $X_{P}$ generated by the perturbation *P*. This would implies the stability of solutions, evolving from initial data of size $\epsilon$, over a times scale of order $O(\epsilon ^{-q} )$. In classical Birkhoff normal form approach the main idea is to construct a symplectic change of coordinates $\Phi$ which transform the Hamiltonian *H* into

$$\begin{aligned} H\circ \Phi =H_0+Z+O(u^{r+2}),\qquad r\gg q, \end{aligned}$$

where $Z$ is in *standard Birkhoff normal form*, i.e. it Poisson commutes with $H_0$. Under suitable non-resonance conditions on the frequencies $\omega _{j}$ one can also ensure that $Z$ Poisson commutes with the *Sobolev norms*

1.5 $$\begin{aligned} \Vert u\Vert _{s}^{2}=\sum _{j\in {\mathbb {Z}}^{d}}(1+|j|^{2})^{\frac{s}{2}}|u_j|^{2}, \end{aligned}$$

which is not a priori guaranteed only by the condition $\{Z,H_0\}=0$. However in this strong non-resonant case, one expect a time of stability of order $O(\epsilon ^{-r})$, since since neither $H_0$ nor $Z$ contribute to the possible *growth* of the Sobolev norm. Of course this is a very favourable situation. General settings are usually more complicated and the strategy described above fails.

Our point of view is the following. First of all, following [5], we decompose the variables in variables of large index (high modes) and variables of small index (low modes), i.e. we split

$$\begin{aligned} u=u^{\le }+u^\perp ,\qquad N\gg 1, \end{aligned}$$

where *N* is a fixed large constant and $u^\perp$ is supported only on $u_{j}$ with indexes $|j|>N$. The first crucial observation is that the terms in the Hamiltonian which are at least *cubic* in high variables $u^\perp$ (the case $q=3$) give a very small contribution. Indeed if *u* is in a space of sufficiently high regularity (say $H^{s}$) one expects a tame-like bound $N^{-s+s_0}$ for the generated vector field (see Lemma 3.8). Therefore as first step we split the Hamiltonian function as

$$\begin{aligned} H=H_0+P_{0}+P_1+P_{2}+P_{\perp }, \end{aligned}$$

where $P_{\perp }$ has a zero of order at least 3 in $u_{\perp }$, where $P_{j}$, $j=0,1,2$ is homogeneuos of degree *j* in $u_{\perp }$. We have the following important remarks:

- First of all we remark that, thanks to the conservation of momentum the monomials (of homogeneity *r*) appearing in the perturbation *P* have the form
  $$\begin{aligned} \begin{aligned}&\Big (\prod _{i=1}^{\ell }u_{j_i}\Big ) \Big (\prod _{i=\ell +1}^{r}\overline{u_{j_i}}\Big )\quad \mathrm{for\; some} \quad 0\le \ell \le r,\\&j_1+\cdots +j_{\ell }-j_{\ell +1}-\cdots -j_{r}=0. \end{aligned} \end{aligned}$$
  All the resonant monomials, i.e. the ones Poisson commuting with $H_0$, are those supported on indexes satisfying
  $$\begin{aligned} \sum _{i=1}^{\ell }\omega _{j_i}-\sum _{k=\ell +1}^{r}\omega _{j_k}=0. \end{aligned}$$
  Hypothesis 2.8 guarantees that the condition above is verified if and only if up to permutation, one has (see (2.22))
  $$\begin{aligned} r=2\ell \quad \textrm{and} \quad \omega _{j_{i}}=\omega _{j_{i+\ell }}, \qquad i=1,\ldots ,\ell . \end{aligned}$$
  This implies that resonant monomials Poisson commute both with $H_0$ and with the Sobolev norm $\Vert \cdot \Vert _{s}^{2}$ in (1.5).
- the term $P_{\perp }$ already gives a small contribution, at least for regular *u*. So we do not apply any normal form procedure to eliminate monomials belonging to $P_{\perp }$.
- By momentum conservation if a homogenous term of degree *q* has only one high variable $u_j$ with $|j|>N$ then one has the bound $|j|\le q N$. This means that these monomials can be eliminate just by requiring the very weak non-resonance condition 1.4. Indeed, in this case, the right hand side of (1.4) can be bounded from below by a constant depending only on *N*. Then no loss of derivatives can arise from these small divisors. Only resonant monomials cannot be eliminated. See the first item for details.
- The crucial point of our strategy is to deal with the terms belonging to $P_{2}$, and here it is fundamental the second assumption on the frequencies $\omega _j$, i.e. they fulfil the *Bourgain’s clustering property*. We refer to Hypothesis 2.5 for a precise statement. Roughly speaking such property implies that the is a partition of $${\mathbb {Z}}^d=\cup _{\alpha }\Omega _\alpha \ $$, made by clusters $\Omega _{\alpha }\subset {\mathbb {Z}}^{d}$ with the following properties: the clusters have a dyadic property that allows to control the $H^{s}$-norm with the $L^{2}$-norm, and indexes $j,k\in {\mathbb {Z}}^{d}$ belonging to different clusters $j\in \Omega _{\alpha }$, $k\in \Omega _{\beta }$, possesses frequencies $\omega _{j}$ and $\omega _{k}$ which are *well-separated*. See formula (2.19).
  Now, consider a monomial of the form
  1.6 $$\begin{aligned} u_{j_1}\overline{u_{j_2}} \Big (\prod _{i=3}^{\ell }u_{j_i}\Big ) \Big (\prod _{i=\ell +1}^{q}\overline{u_{j_i}}\Big )\quad \textrm{with}\quad |j_1|\sim |j_2|\gg \max \{|j_3|,\ldots ,|j_{q}|\}. \end{aligned}$$
  Hypothesis 2.5 guarantees that if the two highest indexes $j_1,j_2$ do not belong to the same cluster then the very weak lower bounds in (1.4) can be improved. This is the content of the fundamental Lemma 3.17. Therefore one can cancel out all the monomials in $P_2$ with the exception of those monomials in (1.6) for which $j_1,j_2$ belong to the same Bourgain’s cluster.

In conclusion, performing a normal form procedure takin into account the remarks above, we transform the Hamiltonian *H* into (see Theorem 3.3)

$$\begin{aligned} \widetilde{H}=H_0+Z_0+Z_{2}+R_{T}+R_{\perp }, \end{aligned}$$

where $R_{\perp }$ is homogeneous of degree at least 3 in $u^\perp$, $R_{T}$ has large minimal degree $O(u^{r})$, $Z_0$ is supported only on *low* modes and commutes both with $H_0$ and $\Vert \cdot \Vert _s^2$, while $Z_2$ is quadratic in the high variables, i.e. it can be seen as a quadratic form in the high variables with coefficients the low variables. In particular it is in block-diagonal normal form (according to Definition 3.2), namely the two highest indexes belong to the same Bourgain’s cluster. The important consequence, proved in Lemma 4.3, is that the flow generated by $Z_2$ is uniformly bounded in $H^{s}$. This follows the ideas implemented in [15, 20, 25] to give upper bounds on the flows of linear Schrödinger equations with multiplicative potential.

## The Abstract Theorem

### Phase Space

Denote ${\mathcal {Z}}^d:={\mathbb {Z}}^d\times \left\{ -1,1\right\}$. Let *g* be a positive definite, symmetric, quadratic form on ${\mathbb {Z}}^d$ and, for $J\equiv (j,\sigma )\in {\mathcal {Z}}^d$, denote

2.1 $$\begin{aligned} \begin{aligned}&\left| J\right| ^2\equiv |j|^2:= \sum _{i = 1}^d |j_i|^2, \quad |J|^2_g \equiv |j|_g^2 := g(j, j). \end{aligned} \end{aligned}$$

We define

2.2 $$\begin{aligned}&\ell ^2_s({\mathcal {Z}}^d;{\mathbb {C}}):= \Big \{ u\equiv (u_J)_{J\in {\mathcal {Z}}^d},\quad u_J\in {\mathbb {C}},\ :\nonumber \\&\left\| u\right\| _s^2:=\sum _{J\in {\mathcal {Z}}^d}\left( 1+\left| J\right| \right) ^{2s}|u_J|^2<\infty \Big \}. \end{aligned}$$

In the following we will simply write $\ell ^2_s$ for $\ell ^2_s({\mathcal {Z}}^d;{\mathbb {C}})$ and $\ell ^2$ for $\ell ^2_0$. We denote by $B_s(R)$ the open ball of radius *R* and center 0 in $\ell ^2_s$. Furthermore in the following ${\mathcal {U}}_s\subset \ell ^2_s$ will always denote an open set containing the origin.

We endow $\ell ^2$ by the symplectic form $\textrm{i}\sum _{j\in {\mathbb {Z}}^d} u_{(j,+)}\wedge u_{(j,-)}$, which, when restricted to $\ell _s^2$ ($s>0$), is a weakly symplectic form.

Correspondingly, given a function $H\in C^1({\mathcal {U}}_s)$, for some *s*, its Hamilton equations are given by

2.3 $$\begin{aligned} \dot{u}_{(j,+)}={-}\textrm{i}\frac{\partial H}{\partial u_{(j,-)}},\qquad \dot{u}_{(j,-)}=\textrm{i}\frac{\partial H}{\partial u_{(j,+)}}, \end{aligned}$$

or, compactly

2.4 $$\begin{aligned} \dot{u}_{(j,\sigma )}={-}\sigma \textrm{i}\frac{\partial H}{\partial u_{(j,{-}\sigma )}}\ . \end{aligned}$$

We will also denote by

2.5 $$\begin{aligned} X_H(u):=(X_J)_{J\in {\mathcal {Z}}^d},\qquad X_{(j,\sigma )}:= {-}\sigma \textrm{i}\frac{\partial H}{\partial u_{(j,{-}\sigma )}} \end{aligned}$$

the corresponding (formal) Hamiltonian vector field.

In the following we will work on the space $\ell ^2_s$ with *s* large. More precisely, all the properties we will ask will be required to hold for all *s* large enough.

### The Class of Functions (and Perturbations)

Given an index $J\equiv (j,\sigma )\in {\mathcal {Z}}^d$ we define the involution

2.6 $$\begin{aligned} {\bar{J}}:=(j,-\sigma ). \end{aligned}$$

Given a multindex $\textbf{J}\equiv (J_1,...,J_r)$, with $J_l\in {\mathcal {Z}}^d$, $l=1,...,r$, we define ${\bar{\textbf{J}}}:=({\bar{J}}_1,...,{\bar{J}}_r)$.

On the contrary, *for a complex number the bar will simply denote the complex conjugate*.

#### Definition 2.1

On $\ell ^2_s$ we define the involution *I* by

2.7 $$\begin{aligned} (Iu)_{J}:=\overline{u_{{\bar{J}}}}. \end{aligned}$$

The sequences such that $Iu=u$ will be called *real sequences*.

Given a multi-index $\textbf{J}\equiv (J_1,...,J_{r})$, we also define its momentum by

2.8 $$\begin{aligned} {\mathcal {M}}(\textbf{J}):=\sum _{l=1}^{r}\sigma _lj_l. \end{aligned}$$

In particular in the following we will deal almost only with multi indexes with zero momentum, so we define

2.9 $$\begin{aligned} {\mathcal {I}}_r:=\left\{ \textbf{J}\in ({\mathcal {Z}}^d)^{r}\ :\ {\mathcal {M}}(\textbf{J})=0 \right\} . \end{aligned}$$

Given a homogeneous polynomial *P* of degree *r*, namely $P:\ell ^2_s\rightarrow {\mathbb {C}}$ for some *s*, it is well known that it can be written in a unique way in the form

2.10 $$\begin{aligned} P(u)=\sum _{J_1,...,J_{r}\in {\mathcal {Z}}^d}P_{J_1,...,J_r}u_{J_1}...u_{J_r}\ , \end{aligned}$$

with $P_{J_1,...,J_{r}}\in {\mathbb {C}}$ symmetric with respect to any permutation of the indexes.

We are now ready to specify the class of functions we will consider.

#### Definition 2.2

(*Polynomials*). Let $r\ge 1$. We denote by ${\mathcal {P}}_r$ the space of formal polynomials *P*(*u*) of the form (2.10) satisfying the following conditions:

1. *(Momentum conservation):* *P*(*u*) contains only monomyals with zero momentum, namely (recall (2.9))
  2.11 $$\begin{aligned} P(u)=\sum _{\textbf{J}\in {\mathcal {I}}_r}P_{\textbf{J}}u_{J_1}...u_{J_r} ; \end{aligned}$$
2. *(Reality):* for any $\textbf{J}\in (\mathcal {Z}^{d})^{r}$, one has $\overline{P_{{\bar{\textbf{J}}}}}=P_{\textbf{J}}$.
3. *(Boundedness):* The coefficients $P_{\textbf{J}}$ are bounded, namely
  $$\begin{aligned} \sup _{\textbf{J}\in {\mathcal {I}}_r}|P_{\textbf{J}}|<\infty . \end{aligned}$$

For $R>0$ we endow the space ${\mathcal {P}}_r$ with the family of norms

2.12 $$\begin{aligned} \left\| P\right\| _R:=\sup _{\textbf{J}\in {\mathcal {I}}_r}|P_{\textbf{J}}|R^{r}. \end{aligned}$$

Given $r_2\ge r_1\ge 1$ we denote by ${\mathcal {P}}_{r_1,r_2}:=\bigcup _{l=r_1}^{r_2}{\mathcal {P}}_l$ the space of polynomials *P*(*u*) that may be written as

$$\begin{aligned} P=\sum _{l=r_1}^{r_2}P_l,\qquad P_{l}\in {\mathcal {P}}_{l}, \end{aligned}$$

endowed with the natural norm

$$\begin{aligned} \left\| P\right\| _R:=\sum _{l=r_1}^{r_2}\left\| P_l\right\| _R. \end{aligned}$$

Of course other possible choices for the norm (2.12) are possible (see for instance the majorant norm on multilinear operators in [17]). However this choice is sufficient to prove the needed properties on the polynomials in $\mathcal {P}_r$. We refer to Sect. 3.1.

#### Remark 2.3

By the reality condition (*P*.2) in Definition 2.2, one can note that if $P\in {\mathcal {P}}_r$ then

- $P(u)\in {\mathbb {R}}$ for all real sequence *u* (see Definition 2.1).
- Fix $J_1,J_2\in {\mathbb {Z}}^d$ and define
  $$\begin{aligned} A_{J_1,J_2}(u):=\sum _{\begin{array}{c} J_3,...,J_r \in {\mathbb {Z}}^d \\ (J_1,J_2,J_3,\ldots ,J_r)\in \mathcal {I}_r \end{array}}P_{J_1,J_2,J_3,...,J_r}u_{J_3}...u_{J_r}. \end{aligned}$$
  Then, for all real sequence *u*, one has
  2.13 $$\begin{aligned} A_{(j_1,+),(j_2,-)}={\bar{A}}_{(j_2,+),(j_1,-)}; \end{aligned}$$
  this “formal selfadjointness” will play a fundamental role in the following.

#### Definition 2.4

(*Functions*). We say that a function $P\in C^{\infty }({\mathcal {U}}_s;{\mathbb {C}})$ belongs to class ${\mathcal {P}}$, and we write $P\in \mathcal {P}$, if

$\bullet$ all the terms of its Taylor expansion at $u=0$ are of class ${\mathcal {P}}_r$ for some *r*;

$\bullet$ the vector field $X_{P}$ (recall (2.5)) belongs to $C^{\infty }({\mathcal {U}}_s;\ell ^2_s)$ for all $s>d/2$.

The Hamiltonian systems that we will study are of the form

2.14 $$\begin{aligned} H=H_0+P, \end{aligned}$$

with $P\in {\mathcal {P}}$ and $H_0$ of the form

2.15 $$\begin{aligned} H_0(u):=\sum _{j\in {\mathbb {Z}}^d} \omega _ju_{(j,+)}u_{(j,-)}, \end{aligned}$$

and $\omega _j\in {\mathbb {R}}$ a sequence on which we are going to make some assumptions in the next subsection.

### Statement of the Main Result

We need the following assumption.

#### Hypothesis 2.5

The frequency vector $\omega =(\omega _{j})_{j\in {\mathbb {Z}}^d}$ satisfies the following.

1. There exist constants $C_1>0$ and $\beta >1$ such that, $\forall j$ large enough one has
  $$\begin{aligned} \frac{1}{C_1}\left| j\right| ^\beta \le \omega _j\le C_1 \left| j\right| ^\beta . \end{aligned}$$
2. For any $r\ge 3$ there exist $\gamma _r>0$ and $\tau _r$ such that the following condition holds for all *N* large enough
  2.16 $$\begin{aligned}&\forall J_1,...,J_r\ \;\;\;\text {with}\ \;\;\; \left| J_l\right| \le N,\ \;\;\forall l=1,...,r \nonumber \\&\qquad \sum _{l=1}^r\sigma _{j_l}\omega _{j_l}\not =0\quad \Longrightarrow \quad \left| \sum _{l=1}^r\sigma _{j_l}\omega _{j_l}\right| \ge \frac{\gamma _r}{N^{\tau _r}}. \end{aligned}$$
3. There exists a partition
  2.17 $$\begin{aligned} {\mathbb {Z}}^d=\bigcup _{\alpha }\Omega _\alpha , \end{aligned}$$
  with the following properties:
  F.3.1
    $*$ either $\Omega _\alpha$ is finite dimensional and centered at the origin, namely there exists $C_1$ such that
      $$\begin{aligned} j\in \Omega _\alpha \,\quad \Longrightarrow \quad |j|\le C_1\, \end{aligned}$$
    $*$ or it is dyadic, namely there exists a constant $C_2$ independent of $\alpha$ such that
      2.18 $$\begin{aligned} \sup _{j\in \Omega _\alpha }\left| j\right| \le C_2\inf _{j\in \Omega _\alpha }\left| j\right| \ . \end{aligned}$$
  F.3.2 There exist $\delta >0$ and $C_3=C_3(\delta )$ such that, if $j\in \Omega _\alpha$ and $i\in \Omega _\beta$ with $\alpha \not =\beta$, then
    2.19 $$\begin{aligned} \left| i-j\right| +\left| \omega _i-\omega _j\right| \ge C_3(\left| i\right| ^\delta +\left| j\right| ^\delta )\ . \end{aligned}$$

#### Remark 2.6

If in the above inequality one substitutes $|i-j|$ by a norm of $\left| i-j\right|$ which is equivalent to the norm |.|, then (2.19) still holds with a different constant. The same is true if one substitutes the norms at right hand side with equivalent norms. In the following we will exploit such a freedom.

Finally, we need a separation property of the resonances, namely that the resonances do not couple very low modes with very high modes. To state this precisely, we first define an equivalence relation on ${\mathbb {Z}}^d$

#### Definition 2.7

For $i,j\in {\mathbb {Z}}^d$, we say that $i\sim j$ if $\omega _i=\omega _j$. We denote by [*i*] the equivalence classes with respect to such an equivalence relation.

#### Hypothesis 2.8

The frequency vector $\omega =(\omega _{j})_{j\in {\mathbb {Z}}^d}$ satisfies the following.

1. The equivalence classes are dyadic, namely there exists $C>0$ such that
  2.20 $$\begin{aligned} C\inf _{j\in [i]}|j|\ge \sup _{j\in [i]}|j|,\quad \forall i\in {\mathbb {Z}}^d; \end{aligned}$$
2. *Non-resonance*: Given any sequence of multiindexes $(j_k,\sigma _k)\in {\mathcal {Z}}^d$, $k=1,\ldots ,l$, one has that the condition
  2.21 $$\begin{aligned} \sum _{i=1}^l\sigma _i\omega _{j_i}=0\, \end{aligned}$$
  implies that $\ell$ is even and that there exists a permutation $\tau$ of (1, ..., *l*) such that
  2.22 $$\begin{aligned} \forall \, i=1,...,l/2 , \quad \omega _{j_{\tau (i)}}=\omega _{j_{\tau (i+l/2)}}\quad \textrm{and} \quad \sigma _{\tau (j)}=\sigma _{\tau (j+l/2)}. \end{aligned}$$
  We say that a sequence of multiindexes satisfying (2.22) is *resonant*, otherwise we say that it is *non-resonant*.

#### Remark 2.9

We point out that the Hypothesis 2.8 is only used in Sect. 4 in order to prove energy estimates for the system in normal form, see Lemma 4.2.

Our main abstract theorem pertains the Cauchy problem

2.23 $$\begin{aligned} \left\{ \begin{aligned}&\dot{u}=X_{H}(u)\\&u(0)=u_0 \end{aligned} \right. \ . \end{aligned}$$

#### Theorem 2.10

Consider the Cauchy problem (2.23) where *H* has the form (2.14) with $H_0$ as in (2.15) and $P\in {\mathcal {P}}$ vanishing at order at least 3 at $u=0$. Assume that the frequencies $\omega _j$ fulfill Hypotheses 2.5, 2.8 and let $\beta >1$ be the constant given by Hyp. 2.5. For any integer *r* there exists $s_r\in {\mathbb {N}}$ such that for any $s\ge s_r$ there exists $\epsilon _0>0$ and $c>0$ with the following property: if the initial datum $u_0\in \ell ^2_s$ is real and small, namely if

2.24 $$\begin{aligned} Iu_0=u_0,\quad \epsilon :=\left\| u_0\right\| _{\ell ^2_s}<\epsilon _0, \end{aligned}$$

then the Cauchy problem (2.23) has a unique solution

$$\begin{aligned} u\in C^0((-T_{\epsilon },T_{\epsilon }),\ell ^2_s)\cap C^1((-T_{\epsilon },T_{\epsilon }),\ell ^2_{s-\beta })\, \end{aligned}$$

with $$T_\epsilon >c\epsilon ^{-r}$$. Moreover there exists $$ C>0$$ such that

2.25 $$\begin{aligned} \sup _{|t|\le T_\epsilon }\left\| u(t)\right\| _{\ell ^2_s}\le C\epsilon . \end{aligned}$$

The main step for the proof of Theorem 2.10 consists in proving a suitable normal form lemma which is given in the next section.

## Normal Form

In the following we will use the notation $a\lesssim b$ to mean there exists a constant *C*, independent of all the relevant parameters, such that $a\le Cb$. If we want to emphasize the fact that the constant *C* depends on some parameters, say *r*, *s*, we will write $a\lesssim _{s,r}b$. We will also write $a\simeq b$ if $a\lesssim b$ and $b\lesssim a$.

Furthermore in order to separate low and high frequency modes in a way coherent with the resonance relations we have to measure the size of the indexes $j\in Z^d$ by the size of the corresponding frequency. Precisely, we define

3.1 $$\begin{aligned} \left| j\right| _{\omega }:=|\omega _j|^{1/\beta },\quad \left| J\right| _{\omega }\equiv \left| (J,\sigma )\right| _{\omega }:=\left| j\right| _{\omega }\ . \end{aligned}$$

### Remark 3.1

In general $\left| .\right| _{\omega }$ is not a norm, since the triangular inequality could fail to hold, however this will not cause any problem in the forthcoming developments.

In the following we will informally say that an index *j* is larger then *N* if $\left| j\right| _{\omega }>N$.

We need the following definition.

### Definition 3.2

(*N-block normal form*). Let $\bar{r}\ge 3$ and $N\gg 1$. We say that a polynomial $Z\in {\mathcal {P}}_{3,{\bar{r}}}$ of the form

$$\begin{aligned} Z=\sum _{l=3}^{{\bar{r}}}\sum _{\textbf{J}\in {\mathcal {I}}_l}Z_{\textbf{J}}u_{J_1}...u_{J_l}\ , \end{aligned}$$

(recall Definition 2.2)

is in *N*-*block normal form* if $Z_{\textbf{J}}\not =0$ *only* if $\textbf{J}\equiv (J_1,...,J_l)$ fulfills **one** of the following two conditions:

1. $\left| J_n\right| _{\omega }\le N$ for any $n=1,\ldots ,l$ and $\sum _{n=1}^l\sigma _{j_n}\omega _{j_n}=0$;
2. there exist *exactly* 2 indexes larger than *N*, say $J_1$ and $J_2$ and the following two conditions hold:
  2.1 $$J_1=(j_1,\sigma _1)$$, $$J_2=(j_2,\sigma _2)$$ with $$\sigma _1\sigma _2=-1$$.
  2.2 there exist $\alpha$ such that $j_1,j_2\in \Omega _\alpha$, namely both the large indexes belong to the same cluster^1^$\Omega _\alpha$.

We now state the main result of this section.

### Theorem 3.3

Fix any $N\gg 1$, $s_0>d/2$ and consider the Hamiltonian (2.14) with $\omega _j$ fulfilling Hypothesis 2.5 and $P\in {\mathcal {P}}$. For any ${\bar{r}}\ge 3$ there are $\tau >0$, $s_{\bar{r}}>s_0$ such that for any $s\ge s_{\bar{r}}$ there exist $R_{s,{\bar{r}}}$, $C_{s,{\bar{r}}}>0$ such that for any $R<R_{s, \bar{r}}$ the following holds. If

3.2 $$\begin{aligned} RN^\tau <R_{s, {\bar{r}}} , \end{aligned}$$

then there exists an invertible canonical transformation

3.3 $$\begin{aligned} {\mathcal {T}}^{({\bar{r}})},\; [{\mathcal {T}}^{({\bar{r}})}]^{-1}\, :B_s(R)\rightarrow B_s(C_{s,{\bar{r}}}R), \end{aligned}$$

such that

3.4 $$\begin{aligned} H^{({\bar{r}})}:=H\circ {\mathcal {T}}^{({\bar{r}})}=H_0+Z^{({\bar{r}})}+{\mathcal {R}}_{T}+{\mathcal {R}}_{\perp } \end{aligned}$$

where

- $Z^{({\bar{r}})}\in {\mathcal {P}}_{3,{\bar{r}}}$ is in *N*-block normal form and fulfills
  3.5 $$\begin{aligned} \left\| Z^{({\bar{r}})}\right\| _R\lesssim _{{\bar{r}}}R^3\ ; \end{aligned}$$
- ${\mathcal {R}}_{T}$ is such that $X_{{\mathcal {R}}_{T}}\in C^{\infty }(B_s(R_{s,{\bar{r}}});\ell ^2_s)$ and
  3.6 $$\begin{aligned} \sup _{\left\| u\right\| _s\le R}\left\| X_{{\mathcal {R}}_{T}}(u)\right\| _s\lesssim _{{\bar{r}},s}R^2 (RN^\tau )^{{\bar{r}}-3},\quad \forall R\le R_{s,{\bar{r}}}\ ; \end{aligned}$$
- ${\mathcal {R}}_{\perp }$ is such that $X_{{\mathcal {R}}_{\perp }}\in C^{\infty }(B_s(R_{s,{\bar{r}}});\ell ^2_s)$ and
  3.7 $$\begin{aligned} \sup _{\left\| u\right\| _s\le R}\left\| X_{{\mathcal {R}}_{\perp }}(u)\right\| _s\lesssim _{ {\bar{r}},s}\frac{R^2}{N^{s-s_0}},\quad \forall R\le R_{s,{\bar{r}}}\ . \end{aligned}$$

The rest of the section is devoted to the proof of this theorem and is split in a few subsections.

### Properties of the Class of Functions ${\mathcal {P}}$

First we give the following lemma.

#### Lemma 3.4

(Estimates on the vector field). Fix $r\ge 3$, $R>0$. Then for any $s> s_0 >d/2$ there exists a constant $C_{r,s}>0$ such that, $\forall P\in {\mathcal {P}}_r$, the following inequality holds:

$$\begin{aligned} \left\| X_{P}(u)\right\| _s\le C_{r,s}\frac{\left\| P\right\| _R}{R} \ ,\qquad \forall \, u\in B_s(R). \end{aligned}$$

#### Proof

Let $P \in {{\mathcal {P}}}_r$. Then (recalling (2.5)) one has $X_P=((X_P)_J)_{J\in {\mathcal {Z}}^d}$ with

3.8 $$\begin{aligned} \begin{aligned} (X_P)_{(j,+)}&={-}\textrm{i}\partial _{u_{(j,-)}} P \\&= {-}\textrm{i}r \sum _{{\begin{array}{c} J_1, \ldots , J_{r - 1} \in \mathcal{Z}^d, \, J=(j,+) \\ \mathcal{M}(J_1, \ldots , J_{r - 1}) + j = 0 \end{array}}} P_{J,J_1,....,J_{r-1}} u_{J_1} \ldots u_{J_{r - 1}} \end{aligned} \end{aligned}$$

and similarly for $(X_P)_{(j,-)}$. Remark that the r.h.s. of (3.8) defines a unique symmetric $(r-1)$-linear form

3.9 $$\begin{aligned} (\widetilde{X_P})_{(j,+)}(u^{(1)},...,u^{(r-1)}):=\textrm{i}r \!\! \sum _{{\begin{array}{c} J_1, \ldots , J_{r - 1} \in \mathcal{Z}^d \\ \mathcal{M}(J_1, \ldots , J_{r - 1}) +j = 0 \end{array}}} P_{J,J_1,....,J_{r-1}} u_{J_1}^{(1)} \ldots u_{J_{r - 1}}^{(r-1)}. \end{aligned}$$

In order to apply Lemma A.1 we decompose

3.10 $$\begin{aligned} u^{(l)}=u_+^{(l)}+u_-^{(l)},\ \text {with}\ u_\sigma ^{(l)}:=(u_{(j,\sigma )}^{(l)})_{j\in {\mathbb {Z}}^d}\ . \end{aligned}$$

Substituting in the previous expression we have

3.11 $$\begin{aligned} \begin{aligned} (\widetilde{X_P})_{+}(u^{(1)}&,...,u^{(r-1)})=\\&=\sum _{l=0}^{r-1} \left( \begin{matrix} r-1\\ l \end{matrix} \right) (\widetilde{X_P})_{+}(u^{(1)}_+,...,u_+^{(l)},u_-^{(l+1)},...,u_-^{(r-1)} )\ . \end{aligned} \end{aligned}$$

Now each of the addenda of (3.11) fulfills the assumptions of Lemma A.1. Therefore, since $\left\| u\right\| _{s_0}\le \left\| u\right\| _{s}$ one has

$$\begin{aligned} \begin{aligned}&\left\| (\widetilde{X_P})_{+}(u^{(1)}_+,...,u_+^{(l)},u_-^{(l+1)},...,u_-^{(r-1)} )\right\| _s\\&\qquad \qquad \qquad \qquad \qquad \lesssim \sup _{\begin{array}{c} (J,J_1,\ldots ,J_r)\in (\mathcal {Z}^{d})^r \end{array}}\left| P_{J,J_1,...,J_{r-1}}\right| \left\| u^{(1)}\right\| _s...\left\| u^{(r-1)}\right\| _s. \end{aligned} \end{aligned}$$

Taking all the $u^{(l)}$ equal to $u\in B_{s}(R)$ (i.e. $\Vert u\Vert _s<R$) and recalling the norm in (2.12) one gets the thesis for $({X_{P}})_+$. Similarly one gets the thesis for $({X_{P}})_-$ and this concludes the proof of the lemma. $\square$

As usual given two functions $f_1,f_2\in C^{\infty }(\ell ^2_s;{\mathbb {C}})$ we define their Poisson Brackets by

3.12 $$\begin{aligned} \left\{ f_1;f_2\right\} := \textrm{i}\sum _{j\in {\mathbb {Z}}^d}\left( \frac{\partial f_1}{\partial u_{(j,-)}}\frac{\partial f_2}{\partial u_{(j,+)}}-\frac{\partial f_1}{\partial u_{(j,+)}}\frac{\partial f_2}{\partial u_{(j,-)}}\right) \equiv df_1 X_{f_2} , \end{aligned}$$

which could be ill defined (but will turn out to be well defined in the cases we will consider).

We recall that if both $f_1$ and $f_2$ have smooth vector field then

3.13 $$\begin{aligned} X_{\{f_1;f_2\}}=[X_{f_1};X_{f_2}], \end{aligned}$$

with $[\cdot ;\,\cdot ]$ denoting the commutator of vector fields.

#### Lemma 3.5

(Poisson brackets). Given two polynomials $P_1\in {\mathcal {P}}_{r_1}$ and $P_2\in {\mathcal {P}}_{r_2}$, one has $\left\{ P_1;P_2\right\} \in {\mathcal {P}}_{r_1+r_2-2}$ with

$$\begin{aligned} \left\| \left\{ P_1;P_2\right\} \right\| _{R}\le \frac{2r_1r_2}{R^2}\left\| P_{r_1}\right\| _{R}\left\| P_{r_2}\right\| _{R}. \end{aligned}$$

#### Proof

It follows by formula (3.12) recalling (2.12) and exploiting the momentum conservation. $\square$

We now fix some large $N>0$, but will track the dependence of all the constants on *N*. Corresponding to *N* we define a decomposition of *u* in *low* and *high* modes. Precisely, we define the projectors

3.14 $$\begin{aligned} \Pi ^{\le }u:=(u_J)_{\left| J\right| _{\omega }\le N},\quad \Pi ^{\perp }u:=(u_J)_{\left| J\right| _{\omega }> N}\ \end{aligned}$$

and denote

3.15 $$\begin{aligned} u^{\le }:=\Pi ^{\le }u,\quad u^{\perp }:=\Pi ^{\perp }u, \end{aligned}$$

so that $$u=u^{\le }+u^\perp $$.

As in [1, 5], a particular role is played by the polynomials $P\in {\mathcal {P}}_r$ which are quadratic or cubic in $u^\perp$. We are now going to give a precise meaning to this formal statement. First, given $f\in C^{\infty }({\mathcal {U}}_s;{\mathbb {C}})$, we denote by

$$\begin{aligned} d^lf(u)(h_1,...,h_l)\ \end{aligned}$$

the *l*-th differential of *f* evaluated at *u* and applied to the increments $$h_1,...,h_l $$.

#### Definition 3.6

Let $P\in {\mathcal {P}}_r$ and recall the notation (3.15).

$\bullet$ We say that *P* has has a zero of order 0 in $u^{\perp }$ if $P(u^{\le })$ is not identically zero for $u\in \ell _{s}^{2}$.

$\bullet$ We say that *P* has has a zero of order at least 1 in $u^{\perp }$ if $P(u^{\le })=0$, $\forall \, u\in \ell _{s}^{2}$.

$\bullet$ We say that *P* has has a zero of order at least $k\ge 2$ in $u^{\perp }$ if

$$\begin{aligned} \begin{aligned}&P(u^{\le })=0,\\&d^lP(\Pi ^{\le }u)(\Pi ^\perp h_1,...,\Pi ^\perp h_l)=0\,\quad \forall \; u,h_1,...,h_l\in \ell ^2_s\,\;\;\; \forall \; l=1,...,k-1\, \end{aligned} \end{aligned}$$

We say that *P* is homogeneous of degree $k\ge 1$ in $u^{\perp }$ if it has a zero of order at least *k*, but not of order at least $k+1$.

We say that *P* is homogeneous of degree 0 if it has a zero of order 0 in $u^{\perp }$ and $P(u)\equiv P(u^{\le })$ for $u\in \ell _{s}^{2}$.

#### Remark 3.7

By the very definition of normal form, one can decompose $Z^{(r)}=Z_0+Z_2$, with $Z_0$ homogeneous of degree zero in $u^\perp$ and $Z_2$ homogeneous of degree 2 in $u^{\perp }$. Furthermore $Z_0$ is in Birkhoff normal form in the classical sense, namely it contains only resonant monomials, i.e. monomials of the form

$$\begin{aligned} u_{J_1}...u_{J_r} , \quad \text {with}\quad \sum _{l}\sigma _l\omega _{j_l}=0\ . \end{aligned}$$

We also remark that, in view of Hypothesis 2.8-(NR.2) such monomials are *super-action preserving.*

#### Lemma 3.8

For all $s> s_0 >d/2$ and all $r\ge 3$, there exists a constant $C_{r,s}>0$ such that the following holds:

(i) if $P\in {\mathcal {P}}_r$ has a zero of order at least 2 in $u^\perp$, then
  $$\begin{aligned} \sup _{\left\| u\right\| _s\le R}\left\| \Pi ^{\le }X_{P}(u)\right\| _s \le \frac{C_{r,s}}{N^{s- s_0}}\frac{\left\| P\right\| _R}{R}\ ; \end{aligned}$$
(ii) if $P\in {\mathcal {P}}_r$ has a zero of order at least 3 in $u^\perp$, then
  $$\begin{aligned} \sup _{\left\| u\right\| _s\le R}\left\| X_{P}(u)\right\| _s\le \frac{C_{r,s}}{N^{s- s_0}}\frac{\left\| P\right\| _R}{R}\ . \end{aligned}$$

#### Proof

Consider first the case (i) and remark that, using the notation (3.10), we have $\left( \Pi ^{\le }X_P(u) \right) _{\pm }=\pm \textrm{i}\nabla _{u_{\pm }^{\le }}P$, so that $\Pi ^{\le }X_P(u)$ has a zero of order 2 in $u^{\perp }$. It follows that both in the case (i) and in the case (ii) we have to estimate a polynomial function *X*(*u*) of the form (3.8) with a zero of second order in $u^{\perp }$. To exploit this fact consider first the $+$ component and consider again the multilinear form $(\widetilde{X})_+$ as in (3.9): we have

$$\begin{aligned} X_+(u)=X_+(u^\perp +u^\le )= \sum _{l=0}^{r-1} \left( \begin{matrix} r-1\\ l \end{matrix} \right) (\widetilde{X})_{+}(\underbrace{u^\perp ,...,u^\perp }_{l-\text {times}}, \underbrace{u^{\le },...,u^{\le }}_{r-1-l-\text {times} }), \end{aligned}$$

but, since $X_+(u)$ has a zero of at least second order in $u^{\perp }$, one has

$$\begin{aligned} X_+(u)= \sum _{l=2}^{r-1} \left( \begin{matrix} r-1\\ l \end{matrix} \right) (\widetilde{X})_{+}(\underbrace{u^\perp ,...,u^\perp }_{l-\text {times}},\underbrace{u^{\le },...,u^{\le }}_{r-1-l-\text {times} })\ . \end{aligned}$$

Consider the first addendum (which is the one giving rise to worst estimates): proceeding as in the proof of Lemma 3.4 one can apply Lemma A.1 and get the estimate

$$\begin{aligned} \begin{aligned}&\left\| (\widetilde{X})_{+}(u^\perp ,u^\perp ,u^{\le },...,u^{\le })\right\| _s \lesssim \sup _{j,j_1,\ldots , j_{r-1}\in {\mathbb {Z}}^d}\left| P_{j,j_1,---,j_{r-1}}\right| \times \\&\quad \quad \qquad \qquad \qquad \times \left( \left\| u^\perp \right\| _s\left\| u^\perp \right\| _{s_0}\left\| u^\le \right\| ^{r-3}_{s_0}+ \left\| u^\le \right\| _s\left\| u^\perp \right\| ^2_{s_0}\left\| u^\le \right\| ^{r-4}_{s_0}\right) , \end{aligned} \end{aligned}$$

but

$$\begin{aligned} \left\| u^\perp \right\| _{s_0}^2=\sum _{\left| J\right| _{\omega }>N}\langle J\rangle ^{2s_0}\left| u_{J}\right| ^2 =\sum _{\left| J\right| _{\omega }>N}\frac{\langle J\rangle ^{2s}\left| u_J\right| ^2}{\langle J\rangle ^{2(s-s_0)}}\lesssim \frac{\left\| u^\perp \right\| _s^2}{N^{2(s-s_0)}}=\frac{\left\| u\right\| _s^2}{N^{2(s-s_0)}}. \end{aligned}$$

Since, by F.1 one has $\left| j\right| _{\omega }\lesssim \left| j\right|$ and therefore $\frac{1}{\langle j\rangle }\lesssim \frac{1}{N}$, it follows

$$\begin{aligned} \begin{aligned} \left\| (\widetilde{X})_{+}(u^\perp ,u^\perp ,u^{\le },...,u^{\le })\right\| _s&\lesssim \sup _{j,j_1,\ldots , j_{r-1}\in {\mathbb {Z}}^d}\left| P_{j,j_1,---,j_{r-1}}\right| \frac{R^{r-1}}{N^{s-s_0}}\\&\lesssim \frac{\left\| P\right\| _R}{R}\frac{1}{N^{s-s_0}}. \end{aligned} \end{aligned}$$

The other cases can be treated similarly. $\square$

### Lie Tranfsorm

Given $G\in {\mathcal {P}}_{r,{\bar{r}}}$, consider its Hamilton equations $\dot{u}=X_G(u)$, which, by Lemma 3.4, are locally well posed in a neighborhood of the origin. Denote by $\Phi _G^t$ the corresponding flow, then we have the following Lemma whose proof is equal to the finite dimensional case.

#### Lemma 3.9

Consider $\bar{r}\ge r_1\ge r\ge 3$ and $s>s_0>d/2$. There exists $C_{r,s}>0$ such that for any $G\in {\mathcal {P}}_{r,r_1}$ and any $R>0$ satisfying

3.16 $$\begin{aligned} \frac{\left\| G\right\| _R}{R}\le \frac{1}{C_{r,s}}, \end{aligned}$$

the following holds. For any $|t|\le 1$ one has $\Phi _G^t(B_s(R))\subset B_s(2R)$ and the estimate

$$\begin{aligned} \sup _{u\in B_s(R)}\left\| \Phi ^t_G(u)-u\right\| \le \left| t\right| C_{r,s}\frac{\left\| G\right\| _R}{R} ,\quad \forall t\ :\ |t|\le 1\ . \end{aligned}$$

#### Definition 3.10

The map $\Phi _G:=\Phi _G^t\Big |_{t=1}$ is called the *Lie transform* generated by *G*.

In order to describe how a function is transformed under Lie transform we define the operator

$$\begin{aligned} ad_G:C^{\infty }({\mathcal {U}}_s,{\mathbb {C}})&\rightarrow C^{\infty }({\mathcal {U}}_s,{\mathbb {C}})\\ f&\mapsto ad_G f:=\left\{ f;G\right\} , \end{aligned}$$

and its *k*-th power $ad_G^kf:=\{ad_{G}^{k-1}f;G\}$ for $k\ge 1$. Also the following Lemma has a standard proof equal to that of the finite dimensional case.

#### Lemma 3.11

Let $\bar{r}\ge r\ge 3$ and $s>s_0>d/2$ and consider $G\in {\mathcal {P}}_{r,{\bar{r}}}$. There exists $C_{r,s}>0$ such that for any $R>0$ satisfying (3.16) the following holds. For any $f\in C^{\infty }(B_s(2R);{\mathbb {C}})$ and any $n\in {\mathbb {N}}$ one has

3.17 $$\begin{aligned} f(\Phi ^t_G(u))=\sum _{k=0}^n\frac{t^k}{k!}(ad_G^kf)(u)+\frac{1}{n!}\int _0^t(t-\tau )^n (ad_G^{n+1}f)\left( \Phi ^\tau _G(u)\right) d\tau , \end{aligned}$$

$\forall u\in B_{s}(R)$ and any *t* with $|t|\le 1$.

From Lemma 3.5 one has the following corollary.

#### Corollary 3.12

Let $G\in {\mathcal {P}}_{r_1,r_2}$, $F\in {\mathcal {P}}_{r_3,r_4}$, with $r_1,r_2,r_3,r_4\le {\bar{r}}$ and $3\le r_1\le r_2$. Let ${\bar{n}}\in {\mathbb {N}}$ be the smallest integer such that $({\bar{n}}+1)(r_1-2)+r_2>{\bar{r}}$. Then there exists $C_{{\bar{r}}}>0$ such that for any $k\le {\bar{n}}$, one has

$$\begin{aligned} \left\| (ad_G)^kF\right\| _R\le \left( \frac{C_{\bar{r}}\left\| G\right\| _{R}}{R^2}\right) ^k \left\| F\right\| _R\ . \end{aligned}$$

A further standard Lemma we need is the following.

#### Lemma 3.13

Let $G\in {\mathcal {P}}_{r_1,r_2}$, $3\le r_1,r_2\le {\bar{r}}$ and let $\Phi _G$ be the Lie transform it generates. Let $R_s$ by the largest value of *R* such that (3.16) holds. Then there exists $C>0$ such that for any $F\in C^{\infty }(B_s(2R_s))$ satisfying

$$\begin{aligned} \sup _{\left\| u\right\| _s\le 2R}\left\| X_F(u)\right\| _s=:C_R<\infty \ ,\quad \forall R<R_s\ . \end{aligned}$$

one has

$$\begin{aligned} \sup _{\left\| u\right\| _s\le R/C}\left\| X_{F\circ \Phi _G}(u)\right\| _s=:2C_R<\infty ,\quad \forall R<R_s\ . \end{aligned}$$

From Lemma 3.11, Corollary 3.12 and Lemma 3.13, one has the following Corollary which is the one relevant for the perturbative construction leading to the normal form lemma.

#### Corollary 3.14

There exists $\mu _0>0$ such that for any $G\in {\mathcal {P}}_{r,{\bar{r}}}$, $3\le r\le {\bar{r}}$, the following holds. If

$$\begin{aligned} \mu :=\frac{C_{{\bar{r}}}\left\| G\right\| _R}{R^2} <\mu _0, \end{aligned}$$

with $C_{{\bar{r}}}$ the constant of Corollary 3.12, then, for any $F\in {\mathcal {P}}_{r_1,{\bar{r}}}$, $r_1\le {\bar{r}}$, one has

$$\begin{aligned} F\circ \Phi _G={\tilde{F}}+{\mathcal {R}}_{F,G}, \end{aligned}$$

with ${\tilde{F}}\in {\mathcal {P}}_{r+r_1-2,{\bar{r}}}$ (${\tilde{F}}\equiv 0$ if $r+r_1-2>{\bar{r}}$) and ${\mathcal {R}}_{F,G}\in C^{\infty }(B_s(R/C);{\mathbb {C}})$ which fulfill the following estimates

$$\begin{aligned}&\left\| {\tilde{F}}\right\| _R\lesssim _{{\bar{r}}} \mu \left\| F\right\| _R\ , \sup _{\left\| u\right\| _s\le R/C} \left\| X_{{\mathcal {R}}_{F,G}}(u)\right\| _{s}\lesssim \frac{\left\| F\right\| _R}{R}\mu ^{{\bar{n}}}, \end{aligned}$$

with ${\bar{n}}$ as in Corollary 3.12 and *C* as in Lemma 3.13.

### Homological Equation

In order to construct the transformation ${\mathcal {T}}^{({\bar{r}})}$ of Theorem 3.3, we will use the Lie transform generated by auxiliary Hamiltonian functions $G_3,...,G_{{\bar{r}}}$, with $G_\ell \in {\mathcal {P}}_{\ell ,{\bar{r}}}$, which in turn will be constructed by solving the homological equation

3.18 $$\begin{aligned} \left\{ H_0;G\right\} +Z=F \end{aligned}$$

with $F\in {\mathcal {P}}_{\ell ,{\bar{r}}}$ a given polynomial of order 2 in $u^\perp$ and *Z* to be determined, but in *N*-block normal form. In order to solve the homological equation we need a nonresonance condition seemingly stronger than (2.16), but which actually follows from *F*.1, *F*.2, *F*.3 of Hypothesis 2.5.

First we remark that (recall Remark 2.6), by F.1, the assumptions F.2 implies

F.2’ For any $r\ge 3$ there exist $\gamma _r>0$ and $\tau _r$ such that the following condition holds for all *N* large enough
  3.19 $$\begin{aligned}&\forall J_1,...,J_r\ \;\;\;\text {with}\ \;\;\; \left| j_l\right| _{\omega }\le N,\ \;\;\forall l=1,...,r \nonumber \\&\qquad \sum _{l=1}^r\sigma _{j_l}\omega _{j_l}\not =0\quad \Longrightarrow \quad \left| \sum _{l=1}^r\sigma _{j_l}\omega _{j_l}\right| \ge \frac{\gamma _r}{N^{\tau _r}}, \end{aligned}$$
  with redefined constants.

Similarly F.3.2 implies

F.3.2’ There exist $\delta >0$ and $C_3=C_3(\delta )$ such that, if $j\in \Omega _\alpha$ and $i\in \Omega _\beta$ with $\alpha \not =\beta$, then
  3.20 $$\begin{aligned} \left| i-j\right| +\left| \omega _i-\omega _j\right| \ge C_3(\left| i\right| _{\omega }^\delta +\left| j\right| _{\omega }^\delta ), \end{aligned}$$

which is the one we will use.

To state the non-resonance condition we need the following definition.

#### Definition 3.15

(*Non resonant multi-indexes*). For $\ell \in {\mathbb {N}}$ and $N\gg 1$ we say that multi-indexes $\textbf{J}=(J_1,...,J_l)\in \mathcal {I}_{l}$ (see (2.9)), with $J_i=(j_i,\sigma _i)\in \mathcal {Z}^{d}$, are *non resonant multi-indexes* if

3.21 $$\begin{aligned} \sum _{i=1}^{l} \sigma _{j_i}\omega _{j_i}\ne 0, \end{aligned}$$

and **one** of the following conditions holds:

1. there is at most one index larger than *N* ;
2. there exist *exactly* 2 indexes larger than *N*, say $J_1$ and $J_2$ with $\sigma _1\sigma _2=1$ ;
3. there exist *exactly* 2 indexes larger than *N*, say $J_1$ and $J_2$ with $\sigma _1\sigma _2=-1$ and such that there exist $\alpha \ne \beta$ such that $j_1\in \Omega _{\alpha }$ and $j_2\in \Omega _\beta$, namely if the two largest indexes are such that $\sigma _1\sigma _2=-1$ then they belong to different clusters.^2^

We denote by ${\mathcal {J}}_l^N$ the subset of $\mathcal {I}_{l}$ of non resonant multi-indexes.

We denote by $\mathcal {S}_l^N$ the subset of $\mathcal {I}_{l}$ made of multi-indexes $\textbf{J}$ such that there exist at least **three** indexes larger than *N*.

#### Remark 3.16

By Definitions 3.2 and 3.15 we notice that an Hamiltonian $Z\in \mathcal {P}_{r}$, $r\ge 3$, of the form (2.11) but supported only on multi-indexes $\textbf{J}\in \mathcal {I}_{r}{\setminus } \big (\mathcal {J}_{r}^{N}\cup \mathcal {S}_{r}^{N}\big )$ is in *N*-block normal form.

#### Lemma 3.17

Assume Hypothesis 2.5 and let $r\in {\mathbb {N}}$. Then there exist $\tau '_r$ and $\gamma '_r>0$, such that for any $3\le p\le r$ and any multi-index $\textbf{J}\in {\mathcal {J}}_p^N$ one has the bound

3.22 $$\begin{aligned} \left| \sum _{l=1}^{p}\sigma _l\omega _{j_l}\right| \ge \frac{\gamma '_r}{N^{\tau '_r}}\ . \end{aligned}$$

#### Proof

Assume that we are in the case (*I*.1) in Definition 3.15. If all the indexes $j_l$ are smaller than *N*, then there is nothing to prove in view of (3.21) and (*F*.2) in Definition 2.5.

Consider now the case where there is only one index, say $J_1$, larger than *N* and the length of the multi-index is $n+1\le r$. The quantity to be estimated is now

3.23 $$\begin{aligned} \left| \sum _{l=2}^n\sigma _l\omega _{j_l}+\sigma _1\omega _{j_1}\right| \ . \end{aligned}$$

By condition *F*.1, one has

$$\begin{aligned} \left| \sum _{l=2}^n\sigma _l\omega _{j_l}\right| \le rN^\beta C_1\,\quad \textrm{and }\quad |\omega _{j_1}|\ge C_1|j_1|^\beta . \end{aligned}$$

Therefore, if

$$\begin{aligned} |j_1|\ge 2(rC_1^2)^{1/\beta }N=:N_1\, \end{aligned}$$

the estimate (3.22) is satisfied. Hence, the estimate on the quantity (3.23) is nontrivial only if all the indexes are smaller than $N_1$. It follows that we can use (2.16) with *N* replaced by $N_1$, getting

$$\begin{aligned} \left| (3.23)\right| \ge \frac{\gamma _r}{N_1^{\tau _r}}=\frac{\gamma _r}{2^{\tau _r} (rC_1^2)^{\tau _r/\beta }N^{\tau _r}}\, \end{aligned}$$

which implies the bound (3.22) by choosing

$$\begin{aligned} \gamma _r'\le \frac{\gamma _r}{2^{\tau _r} (rC_1^2)^{\tau _r/\beta }}. \end{aligned}$$

This concludes the case (*I*.1).

Consider now the case (*I*.2), i.e. when there are two indexes larger than *N*, say $J_1$ and $J_2$ with $\sigma _1\sigma _2=1$. This case is dealt with similarly to the previous case.

We discuss now to the case $\sigma _1\sigma _2=-1$. By condition (*I*.3) in Definition 3.15 there exist $\alpha \not =\beta$ such that $j_1\in \Omega _{\alpha }$ and $j_2\in \Omega _{\beta }$. It follows (recall (F.3) in Hyp. 2.5) that either

3.24 $$\begin{aligned} \left| \omega _{j_1}-\omega _{j_2}\right| \ge C (\left| j_1\right| _{\omega }^{\delta }+\left| j_2\right| _{\omega }^{\delta }) \end{aligned}$$

or

3.25 $$\begin{aligned} \left| {j_1}-{j_2}\right| \ge C (\left| j_1\right| _{\omega }^{\delta }+\left| j_2\right| _{\omega }^{\delta })\ . \end{aligned}$$

for some $C > 0$. Assume for concreteness that $|j_1|\ge |j_2|$ and $\sigma _1=1$, $\sigma _2=-1$.

Consider first the case where (3.24) holds. The quantity to be estimated is

3.26 $$\begin{aligned} \left| \sum _{l=3}^n\sigma _l\omega _{j_l}+\omega _{j_1}-\omega _{j_2}\right| \ . \end{aligned}$$

Notice that (3.24) implies $\left| \omega _{j_1}-\omega _{j_2}\right| \ge C \left| j_1\right| _{\omega }^\delta$ and that we also have

$$\begin{aligned} \left| \sum _{l=3}^n\sigma _l\omega _{j_l}\right| \le (r-2)N^\beta . \end{aligned}$$

Then it follows that (3.22) is automatic if

$$\begin{aligned} \left| j_1\right| _{\omega }\ge \frac{2(r-2)^{1/\delta }}{C}N^{\beta /\delta }=:N_2. \end{aligned}$$

Hence the bound on (3.26) is nontrivial only if all the indexes are smaller than $N_2$. In this case we can apply (2.16) with $N_2$ in place of *N*, getting

$$\begin{aligned} \left| (3.26)\right| \ge \frac{\gamma _r}{N_2^{\tau _r}}= \frac{\gamma _rC^{\tau _r}}{2^{\tau _r}(r-2)^{\tau _r/\delta } N^{\frac{\beta }{\delta }\tau _r} }\, \end{aligned}$$

which is the wanted estimate, in particular with $\tau '_r\ge \frac{\beta }{\delta }\tau _r$.

It remains to bound (3.26) from below with indexes fulfilling (3.25).

By the zero momentum condition we have

$$\begin{aligned} \sum _{l=3}^{n}\sigma _lj_l+j_1-j_2=0\, \end{aligned}$$

but

$$\begin{aligned} {\left| \sum _{l=3}^{n}\sigma _lj_l \right| \le \sum _{l=3}^n|j_l|\le C\sum _{l=3}^n\left| j_l\right| _{\omega }\le CrN} \end{aligned}$$

while

$$\begin{aligned} \left| j_1-j_2\right| \ge C\left| j_1\right| _{\omega }^\delta . \end{aligned}$$

It follows that in our set there are no indexes with $C \left| j_1\right| _{\omega }^\delta >rN$ (otherwise the zero momentum condition cannot be fulfilled), so all the indexes must be smaller than $N_3:=(rN/C)^{1/\delta }$, and again we can estimate (3.26) using (2.16) with *N* substituted by $N_3$, thus getting the thesis. $\square$

#### Lemma 3.18

(Homological equation). Consider the Homological equation (3.18) with $H_0$ as in (2.15) and $\omega _j$ satisfying Hypotheses 2.5 and where $F\in {\mathcal {P}}_{r,{\bar{r}}}$ is a polynomial having a zero of order 2 in $u^\perp$. Then equation (3.18) has solutions $Z\in {\mathcal {P}}_{r,{\bar{r}}}$ and $G\in {\mathcal {P}}_{r,{\bar{r}}}$ where *Z* is in *N*-block normal form, $N\gg 1$ and moreover

3.27 $$\begin{aligned} \left\| Z\right\| _R&\le \left\| F\right\| _R, \end{aligned}$$

3.28 $$\begin{aligned} \left\| G\right\| _R&\le \frac{N^{\tau '_{{\bar{r}}}}}{\gamma '_{{\bar{r}}}}\left\| F\right\| _R\ . \end{aligned}$$

#### Proof

Notice that, denoting $u_{\textbf{J}}:=u_{J_1}...u_{J_r}$ and recalling (3.12), one has

$$\begin{aligned} \begin{aligned} \left\{ H_0;u_{\textbf{J}}\right\}&= \textrm{i}\sum _{j}\frac{\partial H_0}{\partial u_{(j,-)}} \frac{\partial u_{\textbf{J}}}{\partial u_{(j,+)}} - \frac{\partial H_0}{\partial u_{(j,+)}} \frac{\partial u_{\textbf{J}}}{\partial u_{(j,-)}}\\&=\textrm{i}\sum _{j}\omega _ju_{(j,+)} \frac{\partial u_{\textbf{J}}}{\partial u_{(j,+)}} - \omega _ju_{(j,-)} \frac{\partial u_{\textbf{J}}}{\partial u_{(j,-)}}\\&=\textrm{i}\sum _{j}\omega _ju_{\textbf{J}}\left( \sum _{l=1}^{r}\delta _{(j,+),J_l}\omega _j- \delta _{(j,-),J_l}\omega _j\right) \\&=\textrm{i}u_{\textbf{J}}\left( \sum _{l=1}^{r}\delta _{(j,+),(j_l,\sigma _l)}\omega _{j_l}- \delta _{(j,-),(j_l,\sigma _l)} \omega _{j_l} \right) =\textrm{i}u_{\textbf{J}}\sum _{l=1}^{r}\sigma _l\omega _{j_l}\ . \end{aligned} \end{aligned}$$

It follows that, writing

$$\begin{aligned} P=\sum _{\textbf{J}\in {\mathcal {I}}_r}P_{\textbf{J}}u_{\textbf{J}}\, \end{aligned}$$

one can solve the Homological equation (3.18) by defining (recall Definition 3.15)

$$\begin{aligned} Z(u)&:=\sum _{\textbf{J}\in {\mathcal {I}}_r\setminus {\mathcal {J}}_r^N}P_{\textbf{J}}u_{\textbf{J}},\\ G(u)&:=\sum _{\textbf{J}\in {\mathcal {J}}_r^N}\frac{P_{\textbf{J}}}{\textrm{i}\sum _{l=1}^{r}\sigma _l\omega _{j_l}} u_{\textbf{J}}\ . \end{aligned}$$

By Remark 3.16 we have that *Z* is in *N*-block normal form. The estimates (3.27)–(3.28) immediately follow using Lemma 3.17. $\square$

### Proof of the Normal Form Lemma

Theorem 3.3 is an immediate consequence of the forthcoming Lemma 3.19. To introduce it, we first split

$$\begin{aligned} P={\tilde{P}}+{\mathcal {R}}_{T,0}\, \end{aligned}$$

with ${\tilde{P}}\in {\mathcal {P}}_{3,{\bar{r}}}$ and ${\mathcal {R}}_{T,0}$ having a zero of order at least ${\bar{r}}+1$ at the origin. A relevant role will be played by the quantity $\Vert {\tilde{P}}\Vert _{R}$. In order to simplify the notation, we remark that, for *R* sufficiently small there exists $K_{s,{\bar{r}}}$ such that

$$\begin{aligned} \sup _{\left\| u\right\| _s\le R}\left\| X_{{\mathcal {R}}_{T,0}}(u)\right\| _s\le K_{s,{\bar{r}}} \Vert {\tilde{P}}\Vert _{R}R^{\bar{r}-3}\frac{1}{R} . \end{aligned}$$

#### Lemma 3.19

(Iterative lemma). Assume Hypothesis 2.5 and fix ${\bar{r}}\ge 3$. There exists $\mu _{{\bar{r}}}>0$ such that for any $3\le k\le {\bar{r}}$ and any $s>s_0>d/2$ there exist $R_{s,k}>0$, $C_{s,k},\tau >0$ such that for any $R<R_{s,k}$ and any $N\gg 1$ the following holds. If one has

3.29 $$\begin{aligned} \mu :=\frac{\Vert {\tilde{P}}\Vert _R}{R^2}N^{\tau }<\mu _{\bar{r}}, \end{aligned}$$

then there exists an invertible canonical transformation

3.30 $$\begin{aligned} {\mathcal {T}}^{(k)}:B_s(R)\rightarrow B_s(C_{s,k}R), \end{aligned}$$

with

3.31 $$\begin{aligned} {[}{\mathcal {T}}^{(k)}]^{-1}:B_s(R)\rightarrow B_s(C_{s,k}R), \end{aligned}$$

such that

3.32 $$\begin{aligned} H^{(k)}:=H\circ {\mathcal {T}}^{(k)}=H_0+Z^{(k)}+P_k+{\mathcal {R}}_{T,k}+{\mathcal {R}}_{\perp ,k} \end{aligned}$$

where

- $Z^{(k)}\in {\mathcal {P}}_{3,k}$ is in *N*-block normal form and fulfills
  3.33 $$\begin{aligned} \left\| Z^{(k)}\right\| _R\lesssim _{{\bar{r}},k} \Vert {\tilde{P}}\Vert _{R}\ ; \end{aligned}$$
- $$P_k\in {\mathcal {P}}_{k,{\bar{r}}}$$ fulfills
  3.34 $$\begin{aligned} \left\| P_k\right\| _R\lesssim _{{\bar{r}},k} \Vert {\tilde{P}}\Vert _{R}\mu ^{k-3}\,; \end{aligned}$$
- ${\mathcal {R}}_{T,k}$ is such that $X_{{\mathcal {R}}_{T,k}}\in C^{\infty }(B_s(R_{s,k});\ell ^2_s)$ and
  3.35 $$\begin{aligned} \sup _{\left\| u\right\| _s\le R}\left\| X_{{\mathcal {R}}_{T,k}}(u)\right\| _s\lesssim _{\bar{r},k,s} \Vert {\tilde{P}}\Vert _{R}\mu ^{{\bar{r}}-3}\frac{1}{R},\quad \forall R\le R_{s,k}\ ; \end{aligned}$$
- ${\mathcal {R}}_{\perp ,k}$ is such that $X_{{\mathcal {R}}_{\perp ,k}}\in C^{\infty }(B_s(R_{s,k});\ell ^2_s)$ and
  3.36 $$\begin{aligned} \sup _{\left\| u\right\| _s\le R}\left\| X_{{\mathcal {R}}_{\perp ,k}}(u)\right\| _s\lesssim _{\bar{r},k,s}\frac{ \Vert {\tilde{P}}\Vert _{R}}{R}\frac{1}{N^{s-s_0}},\quad \forall R\le R_{s,k}\ . \end{aligned}$$

The proof occupies the rest of the section and is split in a few Lemmas. We reason inductively. First, we consider the Taylor expansion of $P_k$ in $u^\perp$ and we write

3.37 $$\begin{aligned} P_k=P_{k,eff}+R_{k,\perp }, \end{aligned}$$

with $P_{k,eff}$ containing only terms of degree 0, 1 and 2 in $u^\perp$, while $R_{k,\perp }$ has a zero of order at least 3 in $u^\perp$. Then we determine $G_{k+1}$ and $Z_{k+1}$ by solving the homological equation

3.38 $$\begin{aligned} \left\{ H_0;G_{k+1}\right\} +P_{k,eff}=Z_{k+1}, \end{aligned}$$

so that, by Lemma 3.18 and the inductive assumption (3.34), we get

3.39 $$\begin{aligned} \left\| G_{k+1}\right\| _R&\lesssim \left\| P_k\right\| _RN^{\tau }\lesssim R^2 \Vert {\tilde{P}}\Vert _{R}\mu ^{k-2} \end{aligned}$$

3.40 $$\begin{aligned} \left\| Z_{k+1}\right\| _{R}&\lesssim \left\| P_k\right\| _R N^{\tau }\lesssim \Vert {\tilde{P}}\Vert _{R}\mu ^{k-3}. \end{aligned}$$

Consider the Lie transform $\Phi _{G_{k+1}}$ (recall Definition 3.10) generated by $G_{k+1}$. By the estimate (3.39) and the condition (3.29) we have that there is $R_{s,k+1}>0$ such that (3.16) is fulfilled for $R<R_{s,k+1}$. Hence Lemma 3.9 applies and so we deduce that the map $\Phi _{G_{k+1}}$ is well-posed.

We study now $H^{(k)}\circ \Phi _{G_{k+1}}$. To start with we prove the following Lemma.

#### Lemma 3.20

Let $G_{k+1}$ be the solution of (3.38), then one has

3.41 $$\begin{aligned} H_0\circ \Phi _{G_{k+1}}=H_0+Z_{k+1}-P_{k,eff}+\tilde{H}_0+{\mathcal {R}}_{H_0,G_{k+1}}, \end{aligned}$$

with ${\tilde{H}}_{0}\in {\mathcal {P}}_{k+1,{\bar{r}}}$, and, provided $R<R^0_{k+1}$, for some $R^0_{k+1}$, one has

3.42 $$\begin{aligned} \left\| {\tilde{H}}_0\right\| _R\lesssim \mu ^{k-2} \Vert {\tilde{P}}\Vert _{R}\ . \end{aligned}$$

Furthermore, there exists $C_0>0$ such that one has

3.43 $$\begin{aligned} \sup _{\left\| u\right\| _s\le R/C_0}\left\| X_{{\mathcal {R}}_{H_0,G_{k+1}} }(u)\right\| _s\lesssim \mu ^{{\bar{r}}-3}{ \Vert {\tilde{P}}\Vert _{R}}R^{-1}\ . \end{aligned}$$

#### Proof

Let ${\bar{n}}$ be such that $({\bar{n}}+1)(k-2)+k>{\bar{r}}$; using the expansion (3.17) one gets

3.44 $$\begin{aligned} H_0\circ \Phi _{G_{k+1}}&=H_0+\left\{ H_0;G_{k+1}\right\} +\sum _{l=2}^{{\bar{n}}} \frac{ad_{G_{k+1}}^l}{l!}H_0 +{\mathcal {R}}_{H_0,G_{k+1}} \nonumber \\&=H_0+\left\{ H_0;G_{k+1}\right\} +\sum _{l=2}^{{\bar{n}}} \frac{ad_{G_{k+1}}^{l-1}}{l!}\left\{ H_0;G_{k+1}\right\} +{\mathcal {R}}_{H_0,G_{k+1}} \end{aligned}$$

where we can rewrite explicitly the remainder term as

$$\begin{aligned} {\mathcal {R}}_{H_0,G_{k+1}}=\frac{1}{{\bar{n}}!}\int _0^1(1-\tau )^{\bar{n}}\left( ad_{G_{k+1}}^{{\bar{n}}}\left\{ H_0,G_{k+1}\right\} \right) \circ \Phi _{G_{k+1}}^{\tau }d\tau \ . \end{aligned}$$

Since $G_{k+1}$ fulfills the Homological equation one has

$$\begin{aligned} \left\{ H_0,G_{k+1}\right\} =Z_{k+1}-P_{k,eff}\in {\mathcal {P}}_{k,{\bar{r}}}\, \end{aligned}$$

with

$$\begin{aligned} \left\| \left\{ H_0,G_{k+1}\right\} \right\| _R\lesssim \left\| P_k\right\| _R \lesssim \mu ^{k-3} \Vert {\tilde{P}}\Vert _{R}. \end{aligned}$$

Hence, defining ${\tilde{H}}_0$ to be the sum in Eq. (3.44), one has

$$\begin{aligned} \begin{aligned} \left\| {\tilde{H}}_0\right\| _R&\equiv \left\| \sum _{l=2}^{{\bar{n}}} \frac{ad_{G_{k+1}}^{l-1}}{l!}\left\{ H_0;G_{k+1}\right\} \right\| _R\\&\lesssim \sum _{l=2}^{{\bar{n}}}\left( \frac{C\left\| G_{k+1}\right\| _R}{R^2}\right) ^{l-1} \frac{1}{l!}\mu ^{k-3} \Vert {\tilde{P}}\Vert _{R}\lesssim \mu ^{k-3} \Vert {\tilde{P}}\Vert _{R}\mu =\mu ^{k-2} \Vert {\tilde{P}}\Vert _{R}, \end{aligned} \end{aligned}$$

provided *R* is small enough. Analogously one gets

$$\begin{aligned} \sup _{\left\| u\right\| _s\le R/C}\left\| X_{{\mathcal {R}}_{H_0,G_{k+1}}}(u)\right\| _s\lesssim \mu ^{k-3}\frac{ \Vert {\tilde{P}}\Vert _{R}\mu ^{{\bar{n}}}}{R}, \end{aligned}$$

and, since $k+{\bar{n}}\ge {\bar{r}}$ the thesis follows. $\square$

In an analogous way one proves the following simpler Lemma whose proof is omitted.

#### Lemma 3.21

Let $G_{k+1}\in {\mathcal {P}}_{k,{\bar{r}}}$ fulfills the estimate (3.39), then we have

3.45 $$\begin{aligned} \begin{aligned}&P_k\circ \Phi _{G_{k+1}}=P_k+{\tilde{P}}_{k}+{\mathcal {R}}_{P_k,G_{k+1}},\\&Z^{(k)}\circ \Phi _{G_{k+1}}=Z^{(k)}+{\tilde{Z}}^{(k)}+{\mathcal {R}}_{Z^{(k)},G_{k+1}}, \end{aligned} \end{aligned}$$

and the following estimates hold

$$\begin{aligned} \begin{aligned}&\left\| {\tilde{P}}_k\right\| _R\lesssim \Vert {\tilde{P}}\Vert _{R}\mu ^{k-2},\qquad \sup _{\left\| u\right\| _s\le R/C}\left\| X_{{\mathcal {R}}_{P_k,G_{k+1}}}(u)\right\| _s \lesssim \frac{ \Vert {\tilde{P}}\Vert _{R}\mu ^{{\bar{r}}}}{R},\\&\left\| {\tilde{Z}}^{(k)}\right\| _R\lesssim \Vert {\tilde{P}}\Vert _{R}\mu ^{k-2},\qquad \sup _{\left\| u\right\| _s\le R/C}\left\| X_{{\mathcal {R}}_{Z^{(k)},G_{k+1}}}(u)\right\| _s\lesssim \frac{ \Vert {\tilde{P}}\Vert _{R}\mu ^{{\bar{r}}}}{R}\ . \end{aligned} \end{aligned}$$

#### End of the proof of Lemma 3.19

We consider the Lie transform $\Phi _{G_{k+1}}$ generated by $G_{k+1}$ determined by the equation (3.38) and we define

$$\begin{aligned} {\mathcal {T}}^{(k+1)}:={\mathcal {T}}^{(k)}\circ \Phi _{G_{k+1}}. \end{aligned}$$

By estimate (3.39), condition (3.29), taking *R* small enough, we have that Lemma 3.9 applied to $G_{k+1}$ and the inductive hypothesis on $\mathcal {T}^{(k)}$ imply that $\mathcal {T}^{(k+1)}$ satisfies (3.30)–(3.31) with $k\rightsquigarrow k+1$ and some constant $C_{s,k+1}$.

Recalling (3.41), (3.45) we define

$$\begin{aligned}&Z^{(k+1)}=Z^{(k)}+Z_{k+1},\quad P_{k+1}={\tilde{P}}_k+\tilde{Z}^{(k)}+{\tilde{H}}_0\\&{\mathcal {R}}_{T,k+1}={\mathcal {R}}_{H_0,G_{k+1}}+{\mathcal {R}}_{P_k,G_{k+1}}+{\mathcal {R}}_{Z^{(k)},G_{k+1}}+{\mathcal {R}}_{T,k}\circ \Phi _{G_{k+1}}, \\&{\mathcal {R}}_{\perp ,k+1}=R_{\perp ,k}+ {\mathcal {R}}_{\perp ,k}\circ \Phi _{G_{k+1}}\ . \end{aligned}$$

Then the iterative estimates follow from the estimates of Lemmas 3.20 and 3.21. This concludes the proof. $\square$

#### Proof of Theorem 3.3

Condition (3.2) implies (3.29). Then the result follows by Lemma 3.19 taking $k=\bar{r}$. $\square$

An important consequence of Theorem 3.3 is the following.

#### Corollary 3.22

Consider the Hamiltonian (2.14) with $\omega _j$ fulfilling Hypotheses 2.5 and $P\in {\mathcal {P}}$ (see Definition 2.4). For any $r\ge 3$ there exists $N_r>0$, $\tau >0$ and $s_r>d/2$ and a canonical transformation ${\mathcal {T}}_r$ such that for any $s\ge s_r$ there exists $R_s>0$ and $C_s>0$ such that the following holds for any $R<R_{s}$:

(*i*) one has

3.46 $$\begin{aligned} {\mathcal {T}}_r&\in C^{\infty }(B_s(R/C_s);B_s(R)),\quad {\mathcal {T}}_r^{-1}\in C^{\infty }(B_s(R/C_s);B_s(R)), \end{aligned}$$

3.47 $$\begin{aligned} H^{r}&:=H\circ {\mathcal {T}}_r=H_0+Z^r+{\mathcal {R}}^{(r)}, \end{aligned}$$

where

- $Z^r\in {\mathcal {P}}_{3,r}$ is in $N_r$-block normal form according to Definition 3.2;
- ${\mathcal {R}}^{(r)}$ is such that $X_{{\mathcal {R}}^{(r)}}\in C^{\infty }(B_s(R_{s}/C_s);B_s(R_s))$ and
  3.48 $$\begin{aligned} \sup _{\left\| u\right\| _s\le R}\left\| X_{{\mathcal {R}}^{(r)}}(u)\right\| _s\lesssim _{r}R^{{r+1}},\quad \forall R\le R_{s}/C_{s}\ . \end{aligned}$$

(*ii*) Given $u\in B_s(R)$ we write $u=(u^{\le }, u^{\perp })$ according to the splitting (3.14)–(3.15) with *N* replaced by $N_r$ and we set $Z^r=Z_0+Z_2$ (see Remark 3.7) where $Z_0$ is the part independent of $z^{\perp }$ and $Z_{2}$ is the part homogeneous of order 2 in $z^{\perp }$. Then we have

3.49 $$\begin{aligned} \sup _{\left\| u\right\| _s\le R}\left\| \Pi ^{\le }X_{Z_2}(u^{\le }, u^{\perp })\right\| _s\lesssim _{r}R^{r+1}, \quad \forall R\le R_{s}/C_{s}\ . \end{aligned}$$

#### Proof

Let us fix

3.50 $$\begin{aligned} {\bar{r}}=2r-1, \end{aligned}$$

consider $\tau =\tau _r$ given by Lemma 3.17 and fix

$$\begin{aligned} s_{r}=2(s_0+\tau ({\bar{r}}-3)). \end{aligned}$$

We now take $N_r=N$ such that

3.51 $$\begin{aligned} RN^{\tau }\simeq R^{1/2}\ \iff \ N\simeq R^{-1/2\tau }\ . \end{aligned}$$

With this choices the assumption (3.2) holds taking $R<R_s$ with $R_s$ small enough. Then Theorem 3.3 applies with $s\ge s_r$, $N=N_r$ and $\tau =\tau _r$ chosen above. First of all notice that

3.52 $$\begin{aligned} (RN^\tau )^{{\bar{r}}-3}\simeq \frac{1}{N^{s_r-s_0}}\ \iff \ R^{\frac{\bar{r}-3}{2}}\simeq R^{\frac{s_r-s_0}{2\tau }}. \end{aligned}$$

Then formulæ  (3.46)–(3.47) follow by (3.3)–(3.4) setting $\mathcal {R}^{(r)}=\mathcal {R}_{T}+\mathcal {R}_{\perp }$. Then estimate (3.48) follows by (3.6)–(3.7) and (3.52). The estimate (3.49) follows by Lemma 3.8 and the choice of $N=N_r$ in (3.51). $\square$

## Dynamics and Proof of the Main Result

In this section we conclude the proof of Theorem 2.10.

Consider the Cauchy problem (2.23) (with Hamiltonian *H* as in (2.14)) with an initial datum $u_0$ satisfying (2.24) and fix any $r\ge 3$. Recalling Hypotheses 2.5, 2.8, setting

4.1 $$\begin{aligned} \epsilon \simeq R, \end{aligned}$$

then for $s\gg 1$ large enough and $\epsilon$ small enough (depending on *r*), we have that the assumptions of Corollary 3.22 are fulfilled. Therefore we set

$$\begin{aligned} z_0:=\mathcal {T}_r(u_0), \end{aligned}$$

and we consider the Cauchy problem

4.2 $$\begin{aligned} \dot{z}=X_{H^r}(z),\qquad z(0)=z_0, \end{aligned}$$

with $H^{r}$ given in (3.47). By (3.46) we have that the bound (2.25) on the solution *u*(*t*) of (2.23) follows provided we show

4.3 $$\begin{aligned} \left\| z(t)\right\| ^2_s\lesssim _s \left\| z(0)\right\| ^2_s+R^{{r+2}}|t|, \qquad \left| t\right| <T_R \end{aligned}$$

where *z*(*t*) is the solution of the problem (4.2) and where we denoted

4.4 $$\begin{aligned} T_R:=\sup \left\{ |t|\in {\mathbb {R}}^+\ :\ \left\| z(t)\right\| _s<R\right\} , \end{aligned}$$

the (possibly infinite) escape time of the solution from the ball of radius *R*.

The rest of the section is devoted to the proof of the claim (4.3). To do this we now analyze the dynamics of the system (4.2) obtained from the normal form procedure. To this end we write the Hamilton equations in the form of a system for the two variables $(z^{\le },z^{\perp })$ and also split the normal form $Z^r=Z_0+Z_2$ as in item (*ii*) in Corollary 3.22. We get

4.5 $$\begin{aligned} \dot{z}^{\le }&=\Lambda z^{\le }+X_{Z_0}(z^{\le })+\Pi ^{\le } X_{Z_2}(z^{\le }, z^{\perp })+\Pi ^{\le } X_{{\mathcal {R}}^{(r)}}(z^{\le }, z^{\perp }) , \end{aligned}$$

4.6 $$\begin{aligned} \dot{z}^{\perp }&=\Lambda z^{\perp }+\Pi ^{\perp }X_{Z_2}(z^{\le }, z^{\perp })+\Pi ^{\perp } X_{{\mathcal {R}}^{(r)}}(z^{\le }, z^{\perp })\ . \end{aligned}$$

where $\Lambda$ is the linear operator such that $\Lambda z=X_{H_0}(z)$. The key points to analyze the dynamics are the following:

(i) $Z_0$ is in standard Birkhoff normal form, namely it contains only monomyals Poisson commuting with $H_0$;
(ii) by item (*i*) of Lemma 3.8 one has that $\Pi ^{\le } X_{Z_2}(z^{\le }, z^{\perp })$ is a remainder term (see item (*ii*) in Corollary 3.22);
(iii) $\Pi ^{\perp }X_{Z_2}(z^{\le }, z^{\perp })$ is linear in $z^{\perp }$. Furthermore, for any given trial solution $z^{\le }(t)$ it is a time dependent family of linear operators, which by the property (2.13) are Hamiltonian and thus conserve the $L^2$ norm;
(iv) since $Z_2$ is in normal form it leaves invariant the dyadic decomposition $\Omega _\alpha$ on which the $\ell ^2$ norm is equivalent to all the $\ell ^2_s$ norms.

### Remark 4.1

Recalling (2.15) we have that a monomial $u_{J_1}...u_{J_l}$, $J_i=(j_i,\sigma _i)\in {\mathcal {I}}_l$, $i=1,\ldots ,l$ Poisson commutes with the Hamiltonian $H_0$ if and only if condition (2.21) holds true. Therefore, by Hypothesis 2.8-(NR.2), the Hamiltonian $Z_2$ is supported only on monomials with indexes satisfying (2.22).

Formally we split the analysis in a few lemmas. The first is completely standard and provides a priori estimates on the low frequency part $z^{\le }$ of the solution of (4.5).

### Lemma 4.2

There exists $K_1$ such that for any real initial datum $z_0\equiv (z^{\le }_0,z^{\perp }_0)$ for (4.5), (4.6), fulfilling $\left\| z_0\right\| _s\le R/2$ (with *R* small as in (4.1)) the following holds. One has that

4.7 $$\begin{aligned} \left\| z^{\le }(t)\Vert ^2_{s}\le \Vert z^{\le }(0)\right\| _s^2+ K_1R^{r+2}\left| t\right| ,\quad \forall t,\quad \left| t\right| <T_R, \end{aligned}$$

where $T_{R}$ is given in (4.4).

### Proof

For $i\in {\mathbb {Z}}^d$, define the “superaction”

$$\begin{aligned} J_{[i]}:=\sum _{j\in [i]}z_{(j,-)}z_{(j,+)}\equiv \sum _{j\in [i]}\left| z_{(j,-)}\right| ^2\, \end{aligned}$$

where the sum is over the indexes belonging to the equivalence class of [*i*] according to Definition 2.7 and the second equality follows from the reality of *u*. Then, by the property of being in normal form and by properties (NR.1), (NR.2) in Hypothesis 2.8, we have $\left\{ J_{[i]};Z_0\right\} =0$, so that $\dot{J}_{[i]}=\left\{ J_{[i]};Z_2\right\} +\left\{ J_{[i]};{\mathcal {R}}^{(r)}\right\}$. Denote by ${\mathcal {E}}$ the set of all the equivalence classes of Definition 2.7, and, for $e\in {\mathcal {E}}$, denote

$$\begin{aligned} \langle e\rangle :=\inf _{i\in e}\langle i\rangle \end{aligned}$$

and define the norm

$$\begin{aligned} \left| z\right| _s^2:=\sum _{e\in {\mathcal {E}}}\langle e\rangle ^{2s} J_{e}. \end{aligned}$$

By using the dyadic property (2.20), one has that the norm $|\cdot |_s$ is equivalent to the standard one on $\Pi ^{\le }\ell ^2_s$. Thus we have

$$\begin{aligned}&\frac{d}{dt}\left| z^{\le }\right| _s^2=\sum _{e\in {\mathcal {E}}}\langle e\rangle ^{2s} \frac{d}{dt} J_{e} =\sum _{e\in {\mathcal {E}}}\langle e\rangle ^{2s} \left( \left\{ J_{e};Z_2\right\} +\left\{ J_{e};{\mathcal {R}}^{(r)}\right\} \right) \\&\quad = d\left( \left| z^{\le }\right| _s^2\right) \left( X_{Z_2} (z^{\le },z^{\perp })+X_{{\mathcal {R}}^{(r)}} (z^{\le },z^{\perp })\right) \ . \end{aligned}$$

Then by (3.48)–(3.49)

the last quantity is estimated by a constant times $R^{r+2}$. From this, denoting by $K_0$ the constant in the above inequality, one gets

$$\begin{aligned} \left| z^{\le }(t)\right| _{s}^2\le \left| z^{\le }(0)\right| _s^2+ K_0R^{r+2} |t|. \end{aligned}$$

So we have

$$\begin{aligned} \left\| z^{\le }(t)\right\| _{s}^2\le C \left| z^{\le }(t)\right| _{s}^2\le C\left| z^{\le }(0)\right| _s^2 + CK_0R^{r+2}\left| t\right| , \end{aligned}$$

from which, writing $K_1:=K_0C$ one gets the estimate (4.7). $\square$

We now provide a priori estimates on the high frequencies $z^{\perp }$ which evolve according to (4.6).

### Lemma 4.3

Fix $r\gg 1$. There is $s_r$ such that for any $s>s_r$ there exists $K_3=K_3(s)$ such that for any real initial datum $z_0\equiv (z^{\le }_0,z^{\perp }_0)$ for (4.5), (4.6), fulfilling $\left\| z_0\right\| _s\le R/2$ (with *R* small as in (4.1)) the following holds. One has that

4.8 $$\begin{aligned} \left\| z^{\perp }(t)\right\| ^2_s\le K_2\left\| z^{\perp }(0)\right\| _s^2+K_3R^{r+2}|t|,\quad \forall t\ ,\quad \left| t\right| <T_R, \end{aligned}$$

where $T_{R}$ is given by (4.4).

### Proof

First, we denote by ${\mathcal {Z}}(z^{\le }):\Pi ^\perp \ell ^2\rightarrow \Pi ^\perp \ell ^2$ the family of linear operator s.t. $X_{Z_2}(z^{\le },z^{\perp })={\mathcal {Z}}(z^{\le }) z^{\perp }$; We also write ${\mathcal {Z}}(t):={\mathcal {Z}}(z^{\le }(t))$, with $z^{\le }(t)$ the projection on low modes of the considered solution. We now introduce some further notations. For any $z \in \Pi ^{\perp }\ell ^2$, we introduce the projector $\Pi _\alpha$ associated to the block $\Omega _\alpha$ of the partition. More precisely, for any $\alpha$, we define

4.9 $$\begin{aligned} \Pi _\alpha : \Pi ^{\perp }\ell ^2 \rightarrow \Pi ^{\perp }\ell ^2, \quad \Pi _\alpha u:= \left\{ \begin{matrix} z_{(j,\sigma )} &{}\text {if}&{}j\in \Omega _\alpha \\ 0&{}\text {if}&{}j\not \in \Omega _\alpha \end{matrix}\right. \ . \end{aligned}$$

Then any sequence $z \in \Pi ^{\perp }\ell ^2$ can be written as

4.10 $$\begin{aligned} z = \sum _{\alpha } z_\alpha , \quad z_\alpha := \Pi _\alpha u \ . \end{aligned}$$

By the property 2.2 of Definition 3.2, the normal form operator ${{\mathcal {Z}}}(t)$ has a block-diagonal structure, namely it can be written as

4.11 $$\begin{aligned} {{\mathcal {Z}}}(t) = \sum _{\alpha } {{\mathcal {Z}}}_\alpha (t), \quad \mathcal{Z}_\alpha (t) := \Pi _\alpha {{\mathcal {Z}}}(t) \Pi _\alpha \ . \end{aligned}$$

For any block $\Omega _\alpha$, we define

$$\begin{aligned} n(\alpha ) := \textrm{min}_{j \in \Omega _\alpha } \langle j \rangle \end{aligned}$$

and for any $z \in \ell ^2_s$, we define the norm

$$\begin{aligned}{}[ \! [z] \! ]_s := \Big ( \sum _{\alpha } n(\alpha )^{2 s} \Vert z_\alpha \Vert _0\Big )^{\frac{1}{2}}. \end{aligned}$$

By using the dyadic property (2.18), one has that the norm $[ \! [\cdot ] \! ]_s$ is equivalent to the $\ell ^2_s$-norm $\Vert \cdot \Vert _s$.

Consider now the normal form part of equation (4.6), namely

4.12 $$\begin{aligned} \dot{z}^{\perp }=\Lambda z^{\perp }+\Pi ^{\perp }X_{Z_2}(z^{\le }, z^{\perp })\ ; \end{aligned}$$

by (4.9), (4.10), (4.11), it is block diagonal, namely it is equivalent to the decoupled system

$$\begin{aligned} \partial _t z_\alpha (t) = \Lambda z_\alpha (t) + \mathcal{Z}_\alpha (t) z_\alpha (t)\ . \end{aligned}$$

Since ${{\mathcal {Z}}}_\alpha$ is Hamiltonian, one immediately has that

4.13 $$\begin{aligned} \Vert z_\alpha (t) \Vert _{0} = \Vert z_\alpha (t_0) \Vert _{0}, \quad \forall t, t_0 \in [- T_R, T_R], \quad \forall \alpha . \end{aligned}$$

Therefore, for any $t \in [- T_R, T_R]$, for the solution of (4.12) one has

$$\begin{aligned} \begin{aligned} \Vert z(t) \Vert _s^2&{\lesssim _s} \, [ \! [z(t)] \! ]_s^2 \, \lesssim _s \, \sum _{\alpha } n(\alpha )^{2 s} \Vert z_\alpha (t) \Vert _{0}^2\\&{\mathop {\lesssim _s}\limits ^{(4.13)}} \sum _{\alpha } n(\alpha )^{2 s} \Vert z_\alpha (t_0) \Vert _{0}^2 \lesssim _s \, [ \! [z_0] \! ]_s^2\, {\lesssim _s} \Vert z_0\Vert _s^2 , \end{aligned} \end{aligned}$$

so that, denoting by ${\mathcal {U}}(t,\tau )$ the flow map of (4.12), one has

4.14 $$\begin{aligned} \left\| {\mathcal {U}}(t,\tau )z_0\right\| _{s}\le K_2\left\| z_0\right\| _s,\ \forall t\ . \end{aligned}$$

Consider now (4.6). Using Duhamel formula one gets

$$\begin{aligned} z^{\perp }(t)={\mathcal {U}}(t,0)z_0+\int _0^t{\mathcal {U}}(t,\tau )\Pi ^{\perp }X_{{\mathcal {R}}} (z^{\le }(\tau ),z^{\perp }(\tau ))d\tau \, \end{aligned}$$

which, together with (4.14) and using also (3.48), implies

$$\begin{aligned} \frac{d}{dt}\left\| z^{\perp }\right\| _s^2\le K_rR^{r+2}. \end{aligned}$$

We then deduce the estimate (4.8). $\square$

### Conclusion of the proof of Theorem 2.10

By Lemmas 4.2, 4.3 (see estimates (4.7), (4.8)) we have that the claim (4.3) holds. By a standard bootstrap argument one can show that (recall (4.4), (4.1)) $T_{R} > rsim \epsilon ^{-r}$. This implies the thesis. $\square$

## Applications

Let $\textbf{e}_1,...,\textbf{e}_d$ be a basis of ${\mathbb {R}}^d$ and let

5.1 $$\begin{aligned} \Gamma :=\Big \{x\in {\mathbb {R}}^d\ :\ x=\sum _{j=1}^d2\pi n_j\textbf{e}_j,\quad n_j\in {\mathbb {Z}}\Big \} \end{aligned}$$

be a maximal dimensional lattice. We denote ${\mathbb {T}}^d_\Gamma :={\mathbb {R}}^d/\Gamma$.

To fit our scheme it is convenient to introduce in ${\mathbb {T}}^d_\Gamma$ the basis given by $\textbf{e}_1,...,\textbf{e}_d$, so that the functions turn out to be defined on the standard torus $\mathbb {T}^d:=\mathbb {R}^{d}/(2\pi {\mathbb {Z}})^d$, but endowed by the metric $\texttt{g}_{ij}:=\textbf{e}_j\cdot \textbf{e}_i$. In particular the Laplacian turns out to be

5.2 $$\begin{aligned} \Delta _g:=\sum _{l,n=1}^{d}g_{ln}\partial _{x_{l}}\partial _{x_{n}}\ ,\qquad x=(x_1,\ldots ,x_{d})\in {\mathbb {T}}^{d}, \end{aligned}$$

where $g_{ln}$ is the inverse of the matrix $\texttt{g}_{ij}$. The positive definite symmetric quadratic form of equation (2.1) is then defined by

$$\begin{aligned} g(k,k):=\sum _{l,n=1}^{d}g_{ln}k_lk_n,\qquad \forall \, k\in {\mathbb {Z}}^{d}. \end{aligned}$$

The coefficients $g_{ln}$, $l,n=1,\ldots , d$, of the metric *g* above can be seen as parameters that will be chosen in the set we now introduce. We also assume the symmetry $g_{i j} = g_{j i}$ for any $i, j = 1, \ldots , d$, hence we identify the metric *g* with $(g_{i j})_{i \le j}$, namely we identify the space of symmetric metrics with ${\mathbb {R}}^{\frac{d(d + 1)}{2}}$. We denote by $\Vert g \Vert _{2}^2:= \sum _{i, j} |g_{i j}|^2$

### Definition 5.1

Consider the open set

$$\begin{aligned} \begin{aligned} \mathcal {G}_{0}&:=\left\{ \left( g_{ij}\right) _{i\le j}\in {\mathbb {R}}^{\frac{d (d + 1)}{2}}\;: \; \inf _{x \ne 0} \frac{g(x, x)}{|x|^2} > 0 \right\} . \end{aligned} \end{aligned}$$

Fix $\tau _{*}:= \frac{d(d + 1)}{2} +1$ We then define the set of admissible metrics as follows.

$$\begin{aligned} {{\mathcal {G}}}:= \cup _{\gamma > 0} {{\mathcal {G}}}_\gamma \end{aligned}$$

where

$$\begin{aligned} \begin{aligned} {{\mathcal {G}}}_\gamma&:= \Big \{ g \in {{\mathcal {G}}}_0: \Big | \sum _{i \le j} g_{i j} \ell _{i j} \Big | \ge \dfrac{\gamma }{\Big (\sum _{i \le j} |\ell _{i j}| \Big )^{\tau _*}} \\&\qquad \forall \ell \equiv (\ell _{i j})_{i \le j} \in {\mathbb {R}}^{\frac{d(d + 1)}{2}} \setminus \{ 0 \}\Big \} \end{aligned} \end{aligned}$$

### Remark 5.2

The set ${{\mathcal {G}}}_\gamma$ above satisfies a diophantine estimate $|({{\mathcal {G}}}_0 \cap B_R) {\setminus } {{\mathcal {G}}}_\gamma | \lesssim \gamma$ ($B_R$ is the ball in ${\mathbb {R}}^{\frac{d(d + 1)}{2}}$), implying that ${{\mathcal {G}}}$ has full measure in ${{\mathcal {G}}}_0$ (we denote by $| \cdot |$ the Lebesgue measure). We also point out that in Sect. 5.1, we only take the metric $g \in {{\mathcal {G}}}_0$ and we shall use the convolution potential in order to impose the non-resonance conditions. For the other applications, namely in Sects. 5.2, 5.3, 5.4 we shall use that the metric *g* is of the form $g = \beta {\bar{g}}$, with ${\bar{g}}$ in the set of the admissible metrics ${{\mathcal {G}}}$. We then use the parameter $\beta$, in order to verify the non-resonance conditions required.

### Schrödinger Equations with Convolutions Potentials

We consider Schrödinger equations of the form

5.3 $$\begin{aligned} \textrm{i} \partial _t \psi = - \Delta _g \psi + V * \psi + f(|\psi |^2)\psi , \quad x \in {\mathbb {T}}^d \end{aligned}$$

where $\Delta _{g}$ is in (5.2) with $g\in \mathcal {G}_0$ (see Definition 5.1), *V* is a potential, $*$ denotes the convolution and the nonlinearity *f* is of class $C^\infty ({\mathbb {R}},{\mathbb {R}})$ in a neighborhood of the origin and $f(0) = 0$. Equation (5.3) is Hamiltonian with Hamiltonian function

5.4 $$\begin{aligned} H=\int _{{\mathbb {T}}^d}\left( \nabla \psi \cdot \nabla \varphi +\varphi (V*\psi )+F(\psi \varphi )\right) dx \end{aligned}$$

where *F* is a primitive of *f* and $\varphi$ is a variable conjugated to $\psi$. To get equation (5.3) one has to restrict to the invariant manifold $\varphi ={\overline{\psi }}$.

Fix $n\ge 0$ and $R>0$, then the potential *V* is chosen in the space ${\mathcal {V}}$ given by

5.5 $$\begin{aligned} {\mathcal {V}}:=\left\{ V(x)=\frac{1}{|{\mathbb {T}}^d|_g}\sum _{k\in {\mathbb {Z}}^d}{\hat{V}}_ke^{ik\cdot x}\ :\ {\hat{V}}_k\langle k\rangle ^n\in \left[ -\frac{1}{2},\frac{1}{2} \right] ,\ \forall k\in {\mathbb {Z}}^d \right\} , \end{aligned}$$

which we endow with the product probability measure. Here and below $|{\mathbb {T}}^d|_g$ is the measure of the torus induced by the metric *g*.

#### Theorem 5.3

There exists a set $${\mathcal {V}}^{(res)}\subset {\mathcal {V}}$$ with zero measure such that for any $$V\in {\mathcal {V}}\setminus {\mathcal {V}}^{(res)}$$ the following holds. For any $$r\in {\mathbb {N}}$$, there exists $$ s_r>d/2$$ such that for any $$s>s_r$$ there is $$\epsilon _s>0$$ and $$C>0$$ such that if the initial datum for (5.3) belongs to $$H^s$$ and fulfills $$\epsilon :=\left| \psi \right| _s<\epsilon _s$$ then

$$\begin{aligned} \left\| \psi (t)\right\| _s\le C\epsilon ,\quad \text {for all}\,\quad \left| t\right| \le C\epsilon ^{-r}. \end{aligned}$$

We are now going to prove this theorem. To fit our scheme simply introduce the Fourier coefficients

$$\begin{aligned} \psi (x)=\frac{1}{|{\mathbb {T}}^d|_g^{1/2}} \sum _{j\in {\mathbb {Z}}^d}u_{(j,+)}e^{ij\cdot x} , \quad \varphi (x)=\frac{1}{|{\mathbb {T}}^d|_g^{1/2}} \sum _{j\in {\mathbb {Z}}^d}u_{(j,-)}e^{-ij\cdot x}\ . \end{aligned}$$

In these variables the equation (5.3) takes the form (2.4) with $H=H_0+P$, $H_0$ of the form (2.15) with frequencies

5.6 $$\begin{aligned} \omega _j:=\left| j\right| _g^2+{\hat{V}}_j, \end{aligned}$$

and *P* obtained by substituting in the *F* dependent term of the Hamiltonian (5.4). It is easy to see that the perturbation is of class ${\mathcal {P}}$ of Definition 2.4.

In order to apply our abstract Birkhoff normal form theorem, we only need to verify the Hypotheses 2.5, 2.8. The hypothesis (F.1) in Hyp. 2.5 holds trivially with $\beta =2$ using (5.6).

The hypothesis (F.3) follows by the generalization of the Bourgain’s Lemma proved in [15]. Precisely we now prove the following lemma.

#### Lemma 5.4

The assumption (F.3) of Hypothesis 2.5 holds.

#### Proof

Let $\Omega _\alpha$ be the partition of ${\mathbb {Z}}^d$ constructed in Theorem 2.1 of [15]. It satisfies the properties

$$\begin{aligned} \begin{aligned} ||j|_{g}^2 - |j'|_{g}^2|+ {|j-j'|}&\ge C_0 (|j|^\delta + |j'|^\delta ), \qquad j \in \Omega _\alpha , \quad j' \in \Omega _\beta , \quad \alpha \ne \beta ,\\ \max _{j \in \Omega _\alpha } |j|&\lesssim \min _{j \in \Omega _\alpha } |j|, \end{aligned} \end{aligned}$$

for some $C_0 > 0$ and $\delta \in (0, 1)$. Clearly, one has that if $j \in \Omega _\alpha , j' \in \Omega _\beta$ with $\alpha \ne \beta$, one has that

$$\begin{aligned} \begin{aligned} |\omega _j - \omega _{j'}|+ {|j-j'|}&= ||j|_{g}^2 - |j'|_{g}^2 + {\widehat{V}}_j - {\widehat{V}}_{j'}|+ {|j-j'|}\\&\ge ||j|_{g}^2 - |j'|_{g}^2| +{|j-j'|}- 2 \sup _{j \in {\mathbb {Z}}^d} |{\widehat{V}}_j|\\&\ge ||j|_{g}^2 - |j'|_{g}^2| + {|j-j'|}- 1\\&\ge C_0 (|j|^\delta + |j'|^\delta ) - 1 \ge C_0 (|j|^\delta + |j'|^\delta )/2, \end{aligned} \end{aligned}$$

provided $|j|^\delta + |j'|^\delta \ge \frac{2}{C_0}$, which is verified when $|j| + |j'| \ge C(\delta , C_0)$ for some constant $C(\delta , C_0) > 0$. $\square$

It remains to verify conditions (F.2) in Hyp. 2.5 and (NR.1), (NR.2) in Hyp. 2.8.

Given *r* and *N* we define

$$\begin{aligned} {\mathbb {Z}}^d_N:=\left\{ j\in {\mathbb {Z}}^d\ :\ |j|\le N\right\} ,\\ {\mathcal {K}}^r_N:=\left\{ k\in {\mathbb {Z}}^{{\mathbb {Z}}^d_N}\ :\ 0\not =|k|\le r\right\} , \end{aligned}$$

and remark that its cardinality $\#{\mathcal {K}}^r_N\le N^{dr}$. For $k\in {\mathcal {K}}^r_N$, consider

$$\begin{aligned} {\mathcal {V}}^N_k(\gamma ):=\left\{ V\in {\mathcal {V}}\,\ \left| \omega \cdot k\right| <\gamma \right\} . \end{aligned}$$

#### Lemma 5.5

One has

5.7 $$\begin{aligned} \left| {\mathcal {V}}^N_k(\gamma ) \right| \le 2\gamma N^n \end{aligned}$$

with *n* the number in the definition of ${\mathcal {V}}$ in (5.5).

#### Proof

If ${\mathcal {V}}^N_k(\gamma )$ is empty there is nothing to prove. Assume that ${\tilde{V}}\in {\mathcal {V}}^N_k(\gamma )$. Since $k\not =0$, there exists ${\bar{j}}$ such that $k_{{\bar{j}}}\not =0$ and thus $\left| k_{{\bar{j}}}\right| \ge 1$; so we have

$$\begin{aligned} \left| \frac{\partial \omega \cdot k}{\partial {\hat{V}}_{{\bar{j}}}}\right| \ge 1. \end{aligned}$$

It means that if ${\mathcal {V}}^N_k(\gamma )$ is not empty it is contained in the layer

$$\begin{aligned} \left| \widehat{{\tilde{V}}_{{\bar{j}}}}'-{\hat{V}}'_{{\bar{j}}}\right| \le \gamma \, \end{aligned}$$

whose measure is $\gamma \langle {\bar{j}}\rangle ^n\le 2\gamma N^n$. This implies (5.7). $\square$

#### Lemma 5.6

For any *r* there exists $\tau$ and a set ${\mathcal {V}}^{(res)}\subset {\mathcal {V}}$ of zero measure, s.t., if $V\in {\mathcal {V}}\setminus {\mathcal {V}}^{(res)}$ there exists $\gamma >0$ s.t. for all $N\ge 1$ one has

$$\begin{aligned} \left| \omega \cdot k\right| \ge \frac{\gamma }{N^\tau },\ \forall k\in {\mathcal {K}}^r_N\ . \end{aligned}$$

#### Proof

From Lemma 5.5 it follows that the measure of the set

$$\begin{aligned} {\mathcal {V}}^{(res)}(\gamma ):=\bigcup _{N\ge 1} \bigcup _{k\in {\mathcal {K}}^r_N} {\mathcal {V}}^N_k\left( \frac{\gamma }{ N^{dr+2}}\right) \end{aligned}$$

is estimated by a constant times $\gamma$. It follows that the set

$$\begin{aligned} {\mathcal {V}}^{(res)}:=\cap _{\gamma >0}{\mathcal {V}}^{(res)}(\gamma ) \end{aligned}$$

has zero measure and with this definition the lemma is proved. $\square$

We remark that Lemma 5.6 implies that for $V\in {\mathcal {V}}^{(res)}$ the frequencies $\omega _j$ satisfy $\omega _{j}\ne \omega _i$ for any $i\ne j$. So that the equivalence class [*j*] (see Definition 2.7) are composed by the single element $j\in {\mathbb {Z}}^{d}$.

### Beam Equation

In this section we study the beam equation

5.8 $$\begin{aligned} \psi _{tt}+\Delta ^2_g\psi +m\psi =-\frac{\partial F}{\partial \psi }+\sum _{l=1}^d \partial _{x_l}\frac{\partial F}{\partial (\partial _l \psi )}, \end{aligned}$$

with $F(\psi ,\partial _{x_1}\psi ,...,\partial _{x_d}\psi )$ a function of class $C^{\infty }({\mathbb {R}}^{d+1};{\mathbb {R}})$ in a neighborhood of the origin and having a zero of order 2 at the origin.

Introducing the variable $\varphi =\dot{\psi }\equiv \psi _{t}$, it is well known that (5.8) can be seen as an Hamiltonian system in the variables $(\psi ,\varphi )$ with Hamiltonian function

5.9 $$\begin{aligned} H(\psi ,\varphi ):=\int _{{\mathbb {T}}^d}\left( \frac{\varphi ^2}{2} +\frac{\psi (\Delta _{g}^2+m)\psi }{2} +F(\psi ,\partial _1\psi ,...,\partial _d\psi ) \right) dx \ . \end{aligned}$$

In order to fulfill the diophantine non-resonance conditions on the frequencies we need to make some restrictions on the metric *g* whereas, we only require that the mass $m > 0$ is strictly positive. More precisely, we consider ${\bar{g}}$ be a metric in the set of the admissible metrics ${{\mathcal {G}}}$ given in the definition 5.1. We consider a metric *g* of the form

5.10 $$\begin{aligned} g = \beta {\bar{g}}, \quad \beta \in {{\mathcal {B}}} := (\beta _1, \beta _2), \quad 0< \beta _1< \beta _2 < + \infty . \end{aligned}$$

we shall use the parameter $\beta$ in order to tune the resonances and to impose the non-resonance conditions required in order to apply Theorem 2.10. The precise statement of the main theorem of this section is the following one.

#### Theorem 5.7

Let ${\overline{g}} \in {{\mathcal {G}}}$, There exists a set of zero measure ${{\mathcal {B}}}^{(res)}\subset {{\mathcal {B}}}$ such that if $\beta \in {{\mathcal {B}}} \setminus {{\mathcal {B}}}^{(res)}$ then for all $r\in {\mathbb {N}}$ there exist $s_r>d/2$ such that the following holds. For any $s>s_r$ there exist $\epsilon _{rs},c,C$ such that if the initial datum for (5.8) fulfills

5.11 $$\begin{aligned} \epsilon :=\left\| (\psi _0,{\dot{\psi }}_0)\right\| _s:= \left\| \psi _0\right\| _{H^{s+2}}+ \left\| {\dot{\psi }}_0\right\| _{H^{s}}<\epsilon _{sr}, \end{aligned}$$

then the corresponding solution satisfies

5.12 $$\begin{aligned} \left\| (\psi (t),{\dot{\psi }}(t))\right\| _{s}\le C\epsilon ,\quad \text {for}\quad \left| t\right| \le c\epsilon ^{-r}. \end{aligned}$$

We actually state also a corollary which state that there exists a full measure set of metrics (not only constrained to a given direction ${\overline{g}}$) for which the statements of Theorem 5.7 hold. Let $0< \beta _1 < \beta _2$ and define

5.13 $$\begin{aligned} {{\mathcal {G}}}_0(\beta _1, \beta _2) := \Big \{ g \in {{\mathcal {G}}}_0 : \beta _1 \le \Vert g \Vert _2 \le \beta _2 \Big \}. \end{aligned}$$

where ${{\mathcal {G}}}_0$ is given in the definition 5.1.

#### Corollary 5.8

There exists a zero measure set ${{\mathcal {G}}}^{(res)}_{\beta _1, \beta _2} \subseteq {{\mathcal {G}}}_0(\beta _1, \beta _2)$ such that for any $g \in {{\mathcal {G}}}_0(\beta _1, \beta _2) {\setminus } {{\mathcal {G}}}^{(res)}_{\beta _1, \beta _2}$ the conclusion of theorem 5.7 hold.

#### Proof of Corollary 5.8

To shorten notations in this proof, we denote by $n:= \frac{d(d + 1)}{2}$. For any $\beta _1 \le \beta \le \beta _2$, we denote by $\sigma _\beta$ the surface $n - 1$ dimensional measure on the sphere $\partial B_\beta := \{\Vert g \Vert _2 = \beta \}$. We now prove the following two claims

- **Claim 1.** One has that the surface measure of all diophantine metrics ${{\mathcal {G}}}$ in ${{\mathcal {G}}}_0$ with norm equal 1 has full surface measure in ${{\mathcal {G}}}_0 \cap \partial B_1$, namely $\sigma _1({{\mathcal {G}}} \cap \partial B_1) = \sigma _1({{\mathcal {G}}}_0 \cap \partial B_1)$.
- **Claim 2.** Let ${\overline{g}} \in {{\mathcal {G}}} \cap \partial B_1$ and let ${{\mathcal {B}}}_{{\overline{g}}} \subset (\beta _1, \beta _2)$ the full measure set provided in Theorem 5.7. We shall prove that
  $$\begin{aligned} {{\mathcal {G}}}_{\beta _1, \beta _2}^{(nr)}:= \bigcup _{{\overline{g}} \in \partial B_1 \cap {{\mathcal {G}}}} {{\mathcal {B}}}_{{\overline{g}}} \end{aligned}$$
  has full measure in ${{\mathcal {G}}}_0(\beta _1, \beta _2)$.
  Proof of Claim 1. Let $E \subset \partial B_1$. Then the set
  $$\begin{aligned} \beta E:= \big \{ \beta x: x \in E \big \} \subset \partial B_\beta \end{aligned}$$
  and, by standard scaling properties,
  5.14 $$\begin{aligned} \sigma _\beta (\beta E) = C_n \beta ^{n - 1} \sigma _1(E)\quad \text {for some constant} \quad C_n > 0. \end{aligned}$$
  By (5.13) and Remark (5.2), the set ${{\mathcal {G}}}_{\beta _1, \beta _2}:= {{\mathcal {G}}}_0(\beta _1, \beta _2) \cap {{\mathcal {G}}}$ has full measure in the open set ${{\mathcal {G}}}_0(\beta _1, \beta _2)$. By Fubini one has
  5.15 $$\begin{aligned} |{{\mathcal {G}}}_0(\beta _1, \beta _2)|&= \int _{\beta _1}^{\beta _2} \sigma _{\beta }({{\mathcal {G}}}_0 \cap \partial B_\beta )\, d \beta \nonumber \\&{\mathop {=}\limits ^{(5.14)}} C_n \int _{\beta _1}^{\beta _2} \beta ^{n - 1} \sigma _1({{\mathcal {G}}}_0 \cap \partial B_1)\, d \beta \nonumber \\&= \frac{C_n(\beta _2^n - \beta _1^n)}{n} \sigma _1\Big ( {{\mathcal {G}}}_0 \cap \partial B_1 \Big ) \end{aligned}$$
  and similarly
  5.16 $$\begin{aligned} |{{\mathcal {G}}}_{\beta _1, \beta _2}|&= \int _{\beta _1}^{\beta _2} \sigma _{\beta }({{\mathcal {G}}} \cap \partial B_\beta )\, d \beta \nonumber \\&{\mathop {=}\limits ^{(5.14)}} C_n \int _{\beta _1}^{\beta _2} \beta ^{n - 1} \sigma _1({{\mathcal {G}}} \cap \partial B_1)\, d \beta \nonumber \\&= \frac{C_n(\beta _2^n - \beta _1^n)}{n} \sigma _1\Big ( {{\mathcal {G}}}\cap \partial B_1 \Big ). \end{aligned}$$
  Since $|{{\mathcal {G}}}_0(\beta _1, \beta _2)| = |{{\mathcal {G}}}_{\beta _1, \beta _2}|$, by comparing (5.15), (5.16), one immediately gets that $\sigma _1({{\mathcal {G}}} \cap \partial B_1) = \sigma _1({{\mathcal {G}}}_0 \cap \partial B_1)$.
  Proof of claim 2. By Fubini, the Lebesgue measure $|{{\mathcal {G}}}_{\beta _1, \beta _2}^{(nr)}|$ is
  $$\begin{aligned} \begin{aligned} |{{\mathcal {G}}}_{\beta _1, \beta _2}^{(nr)}|&= \int _{{{\mathcal {G}}} \cap \partial B_1} |{{\mathcal {B}}}_{{\overline{g}}}|\, d \sigma _1({\overline{g}}) = (\beta _2 - \beta _1) \int _{{{\mathcal {G}}} \cap \partial B_1}\, d \sigma _1({\overline{g}}) = (\beta _1 - \beta _2) \sigma _1({{\mathcal {G}}} \cap \partial B_1) \\&{\mathop {=}\limits ^{Claim\, 1}} (\beta _1 - \beta _2) \sigma _1(\partial B_1 \cap {{\mathcal {G}}}_0) = |{{\mathcal {G}}}_0(\beta _1, \beta _2)|. \end{aligned} \end{aligned}$$
  The claimed statement has then been proved.

$$\square $$

To prove Theorem 5.7 we first show how to fit our scheme and then we prove that the Hypotheses of Theorem 2.10 are verified.

To fit our scheme we first introduce new variables

5.17 $$\begin{aligned} u_+(x)&:=\frac{1}{\sqrt{2}}\left( \left( \Delta _{g}^2+m\right) ^{1/4}\varphi +i \left( \Delta _{g}^2+m\right) ^{-1/4}\psi \right) ,\quad \end{aligned}$$

5.18 $$\begin{aligned} u_-(x)&:=\frac{1}{\sqrt{2}}\left( \left( \Delta _{g}^2+m\right) ^{1/4}\varphi -i \left( \Delta _{g}^2+m\right) ^{-1/4}\psi \right) , \end{aligned}$$

and consider their Fourier series, namely, for $\sigma =\pm 1$

$$\begin{aligned} u_\sigma (x)=\frac{1}{|{\mathbb {T}}^d|_g^{1/2}} \sum _{j\in {\mathbb {Z}}^d}u_{(j,\sigma )}e^{\sigma ij\cdot x} \ . \end{aligned}$$

In these variables the beam equation (5.8) takes exactly the form (2.4) with $H=H_0+P$, $H_0$ of the form (2.15) with frequencies

5.19 $$\begin{aligned} \omega _j:=\sqrt{\left| j\right| ^4_g+m}\ . \end{aligned}$$

and *P* obtained by substituting (5.17)–(5.18) in the *F* dependent term of the Hamiltonian (5.9). Thanks to the regularity assumption on *F*, it is easy to see that the perturbation *P* is of class ${\mathcal {P}}$.

The verification of (F.3) in Hyp. 2.5 goes exactly as in the case of the Schrödinger equation, since the asymptotic of $\omega _j$ in (5.19) is $\omega _j = |j|^2_g + O(1)$. The asymptotic condition (F.1) is also trivially fulfilled with $\beta =2$. The main point is to verify the non-resonance conditions (F.2) and the conditions (NR.1), (NR.2) in Hyp. 2.8. This will occupy the rest of this subsection.

First of all we remark that the equivalence classes of Definition 2.7 are simply defined by

$$\begin{aligned} {[}j]\equiv \left\{ i\in {\mathbb {Z}}^d\ :\ |i|_g=|j|_g\right\} \ . \end{aligned}$$

Now, recall that $g = \beta {\overline{g}}$ with ${\overline{g}} \in {{\mathcal {G}}}$ and $\beta \in {{\mathcal {B}}} = [\beta _1, \beta _2]$. One can easily verify that

5.20 $$\begin{aligned} |j|_g = \beta |j|_{{\overline{g}}} \end{aligned}$$

implying that $|j|_g = |k|_g$ if and only if $|j|_{{\overline{g}}} = |k|_{{\overline{g}}}$. Hence the equivalence class [*j*] is

$$\begin{aligned} {[}j]\equiv \left\{ i\in {\mathbb {Z}}^d\,\ |i|_{{\bar{g}}}=|j|_{{\bar{g}}}\right\} . \end{aligned}$$

We are going to prove the following Lemma

#### Lemma 5.9

Let ${\overline{g}} \in {{\mathcal {G}}}$. There exists a set ${{\mathcal {B}}}^{(res)}\subset {{\mathcal {B}}}$ of zero measure, s.t., if $\beta \in {{\mathcal {B}}} {\setminus } {{\mathcal {B}}}^{(res)}$ then (NR.1), (NR.2) and (F.2) hold for the metric $g = \beta \, {\overline{g}}$.

We first need a lower bound on the distance between points with different modulus in ${\mathbb {Z}}^d$. The following lemma holds

#### Lemma 5.10

Fix any $N>1$ and let ${\overline{g}} \in {{\mathcal {G}}}$, $\beta \in {{\mathcal {B}}} = (\beta _1, \beta _2)$, $g = \beta {\overline{g}}$. One has that if $j, k \in {\mathbb {Z}}^d$ such that $|\ell |, |k| \le N$, $|j|_g \ne |k|_g$, then there exists $\gamma > 0$ and a constant $C (\beta _1)$ such that

$$\begin{aligned} ||k|_g^2 - |\ell |_g^2| \ge \frac{C(\beta _1)\gamma }{N^{2\tau _*}} \end{aligned}$$

where $\tau _*$ is given in the definition 5.1.

#### Proof

By recalling the Definition 5.1 of the admissible set ${{\mathcal {G}}}$, since ${\overline{g}} \in {{\mathcal {G}}}$, one has that there exists $\gamma > 0$ such that

$$\begin{aligned} \Big | \sum _{i \le j} {\overline{g}}_{i j} \ell _{i j} \Big | \ge \dfrac{\gamma }{\Big ( \sum _{i \le j} |\ell _{i j}|\Big )^\tau } \quad \forall \ell = (\ell _{i j})_{i \le j} \in {\mathbb {R}}^{\frac{d (d + 1)}{2}} \setminus \{ 0 \}. \end{aligned}$$

By (5.20), since $g = \beta {\overline{g}}$, one has that

5.21 $$\begin{aligned} |\left| k\right| _g^2-\left| \ell \right| _g^2 |&= \beta \Big | \sum _{ij}{\overline{g}}_{ij}\left( k_i k_j- \ell _i \ell _j\right) \Big | \nonumber \\&\ge \beta _1 \dfrac{\gamma }{\Big ( \sum _{i , j} |k_i k_j - \ell _i \ell _j| \Big )^\tau }. \end{aligned}$$

Since $|\ell |, |k| \le N$, one has the following chain of inequalities:

$$\begin{aligned} \begin{aligned} \sum _{i, j} |k_i k_j - \ell _i \ell _j|&\le \sum _{i j} (|\ell _i| |\ell _j| + |k_i| |k_j| ) \lesssim |\ell |^2 + |k|^2 {\mathop {\lesssim }\limits ^{|\ell |, |k| \le N}} N^2. \end{aligned} \end{aligned}$$

The latter inequality, together with (5.21) imply that there exists a constant $C(\beta _1)$ such that

$$\begin{aligned} |\left| k\right| _g^2-\left| \ell \right| _g^2| \ge \dfrac{C(\beta _1) \gamma }{N^{2 \tau }} \end{aligned}$$

uniformly on $\beta \in (\beta _1, \beta _2)$. The claimed statement has then been proved. $\square$

Using the property (5.20), one can easily verify that the frequencies $\omega _j \equiv \omega _j(\beta )$ assume the form

5.22 $$\begin{aligned} \omega _j(\beta ) = \beta ^2 \Omega _j, \quad \Omega _j := \sqrt{|j|^4_{{\overline{g}}} + \frac{m}{\beta ^4}}. \end{aligned}$$

Since $$\beta _2 \ge \beta \ge \beta _1 > 0$$,

$$\begin{aligned} \Big |\sum _{i = 1}^r \sigma _i \omega _{j_i} \Big | = \beta ^2 \Big | \sum _{i = 1}^r \sigma _i \Omega _{j_i}\Big | \ge \beta _1^2 \Big | \sum _{i = 1}^r \sigma _i \Omega _{j_i}\Big | \end{aligned}$$

one can verify non resonance conditions on $\Omega _j$. Since the map

5.23 $$\begin{aligned} (\beta _1, \beta _2) \rightarrow (\zeta _1, \zeta _2) := \Big (\frac{m}{\beta _2^4}, \frac{m}{\beta _1^4} \Big ), \qquad \beta \mapsto \zeta := \frac{m}{\beta ^4} \end{aligned}$$

is an analytic diffeomorphism, we can introduce $\zeta = m/ \beta ^4$ as parameter in order to tune the resonances. Hence we verify non resonance conditions on the frequencies

5.24 $$\begin{aligned} \Omega _j (\zeta ) = \sqrt{|j|_{{\bar{g}}}^4 + \zeta }, \quad j \in {\mathbb {Z}}^d. \end{aligned}$$

#### Lemma 5.11

Let ${\bar{g}} \in {{\mathcal {G}} }$. For any $K\le N$, consider *K* indexes ${j_{1}},...,{j_{K}}$ with $\left| {j_{1}}\right| _g<...\left| {j_{K}}\right| _g\le N$; and consider the determinant

$$\begin{aligned} D:=\left| \begin{matrix} \Omega _{{j_{1}}}&{} \Omega _{{j_{2}}}&{}.&{} .&{}.&{}\Omega _{{j_{K}}}\\ \partial _\zeta \Omega _{{j_{1}}} &{}\partial _\zeta \Omega _{{j_{2}}} &{} .&{} .&{}.&{}\partial _\zeta \Omega _{{j_{K}}} \\ .&{} .&{} .&{} .&{} .&{}. \\ .&{} .&{} .&{} .&{} .&{}. \\ \partial _\zeta ^{K - 1} \Omega _{{j_{1}}}&{} \partial _\zeta ^{K - 1} \Omega _{{j_{2}}}&{} .&{} .&{}.&{}\partial _\zeta ^{K - 1} \Omega _{{j_{K}}} \end{matrix} \right| \end{aligned}$$

There exists $$C>0$$ s.t.

$$\begin{aligned} D \ge \frac{C}{N^{\eta K^2}}, \end{aligned}$$

for some constant $\eta \equiv \eta _d > 0$ depending only on the dimension *d*.

The proof was given in [2]. For sake of completeness we insert it.

#### Proof

For any $i = 1, \ldots , K$, for any $n = 0, \ldots , K - 1$, one computes

$$\begin{aligned} \partial _\zeta ^n \Omega _{j_i}(\zeta ) = C_n (|j_i|_{{\bar{g}}}^4 + \zeta )^{\frac{1}{2} - n} \end{aligned}$$

for some constant $C_n \ne 0$. This implies that

$$\begin{aligned} D \ge C \prod _{i = 1}^K \sqrt{|j_i|_{{\bar{g}}}^4 + \zeta } |\textrm{det}(A)| \end{aligned}$$

where the matrix *A* is defined as

$$\begin{aligned} A = \begin{pmatrix} 1&{} 1 &{}.&{}.&{}.&{}1 \\ x_1&{}x_2&{}.&{}.&{}.&{}x_K \\ .&{}.&{}.&{}.&{}.&{}. \\ .&{}.&{}.&{}.&{}.&{}. \\ x_1^{K - 1}&{} x_2^{K - 1}&{}.&{}.&{}.&{}x_K^{K - 1} \end{pmatrix} \end{aligned}$$

where

$$\begin{aligned} x_i:= \frac{1}{|j_i|_{{\bar{g}}}^4 + \zeta }, \quad i = 1, \ldots , K. \end{aligned}$$

This is a Van der Monde determinant. Thus we have

$$\begin{aligned} \begin{aligned} |\textrm{det}(A)|&= \prod _{1 \le i< \ell \le K} |x_i - x_\ell | = \prod _{1 \le i< \ell \le K} \Big | \frac{1}{|j_i|_{{\bar{g}}}^4 + \zeta } - \frac{1}{|j_\ell |_{{\bar{g}}}^4 + \zeta } \Big | \\&\ge \prod _{1 \le i< \ell \le K} \frac{||j_i|_{{\bar{g}}}^4 - |j_\ell |_{{\bar{g}}}^4|}{||j_i|_{{\bar{g}}}^4 + \zeta | ||j_\ell |_{{\bar{g}}}^4 + \zeta |} \\&\ge \prod _{1 \le i < \ell \le K} \frac{(|j_i|_{{\bar{g}}}^2 + |j_\ell |_{{\bar{g}}}^2) ||j_i|_{{\bar{g}}}^2 - |j_\ell |_{{\bar{g}}}^2|}{||j_i|_{{\bar{g}}}^4 + \zeta | ||j_\ell |_{{\bar{g}}}^4 + \zeta |} \\&{\mathop {\ge }\limits ^{Lemma \,\,5.10}} C N^{- K^2(\tau _* + 4)} \end{aligned} \end{aligned}$$

which implies the thesis. $\square$

Exploiting this Lemma, and following step by step the proof of Lemma 12 of [2] one gets

#### Lemma 5.12

Let ${\overline{g}} \in {{\mathcal {G}}}$. Then and for any *r* there exists $\tau \equiv \tau _r$ with the following property: for any positive $\gamma$ small enough there exists a set $I_\gamma \subset (\zeta _1, \zeta _2)$ such that $\forall \zeta \in I_\gamma$ one has that for any $N\ge 1$ and any multi-index $J_1,...,J_r$ with $|J_l|\le N$ $\forall l$, one has

$$\begin{aligned} \sum _{l=1}^r\sigma _l\Omega _{j_l}\not =0\quad \Longrightarrow \quad \left| \sum _{l=1}^r\sigma _l\Omega _{j_l}\right| \ge \frac{\gamma }{N^{\tau }}\ . \end{aligned}$$

Moreover,

$$\begin{aligned} \left| (\zeta _1, \zeta _2) \setminus I_\gamma \right| \le C \gamma ^{1/r}\ . \end{aligned}$$

#### End of the proof of Lemma 5.9

Let $\gamma > 0$. By recalling the diffeomorphism (5.23), one has that the set

$$\begin{aligned} {{\mathcal {I}}}_\gamma := \{ \beta \in [\beta _1, \beta _2]: m/\beta ^4 \in I_\gamma \}. \end{aligned}$$

satisfies the estimate

$$\begin{aligned} |(\beta _1, \beta _2) \setminus {{\mathcal {I}}}_\gamma | \lesssim \gamma ^{\frac{1}{r}} \end{aligned}$$

Now, if we take $\beta \in {{\mathcal {I}}}_\gamma$ and if $\sum _{i=1}^r\sigma _i\omega _{j_i}\not =0$, one has that

$$\begin{aligned} \begin{aligned} \Big |\sum _{i = 1}^r \sigma _i \omega _{j_i} \Big |&= \beta ^2 \Big | \sum _{i = 1}^r \sigma _i \Omega _{j_i}\Big | {\mathop {\ge }\limits ^{\beta _1 \le \beta \le \beta _2}}\beta _1^2 \Big | \sum _{i = 1}^r \sigma _i \Omega _{j_i}\Big | \\&\ge \frac{\beta _1^2 \gamma }{N^\tau }. \end{aligned} \end{aligned}$$

By the above result, one has that, if

$$\begin{aligned} \beta \in \bigcup _{\gamma >0}{{\mathcal {I}}}_\gamma \, \end{aligned}$$

then (NR.2) holds and furthermore $\bigcup _{\gamma >0}{{\mathcal {I}}}_\gamma$ has full measure. Hence the claimed statement follows by defining ${{\mathcal {B}}}^{(res)}:= {{\mathcal {B}}} {\setminus } \Big ( \bigcup _{\gamma >0}{{\mathcal {I}}}_\gamma \Big )$. $\square$

### The Quantum Hydrodinamical System

We consider the following quantum hydrodynamic system on an irrational torus ${\mathbb {T}}^d_\Gamma$

QHD $$\begin{aligned} \left\{ \begin{aligned}&\partial _{t}\rho =-\texttt{m}\Delta _g\phi -\textrm{div}(\rho \nabla _g\phi )\\&\partial _{t}\phi =-\tfrac{1}{2}|\nabla _g\phi |^{2}- p(\texttt{m}+\rho ) +\frac{\kappa }{\texttt{m}+\rho }\Delta _g\rho -\frac{\kappa }{2(\texttt{m}+\rho )^{2}} |\nabla _g\rho |^{2}, \end{aligned}\right. \end{aligned}$$

where $\texttt{m}>0$, $\kappa >0$, the function *p* belongs to $C^{\infty }(\mathbb {R}_{+};\mathbb {R})$ and $p(\texttt{m})=0$. The function $\rho (t,x)$ is such that $\rho (t,x)+\texttt{m}>0$ and it has zero average in *x*. The variable *x* is on the irrational torus ${\mathbb {T}}^d$ (as in the previous two applications). We assume the conditions

5.25 $$\begin{aligned} p'(\texttt{m})>0. \end{aligned}$$

We shall use Theorem 2.10 in order to prove the following almost global existence result. In order to give a precise statement of the main result, we shall introduce the following notation. Given a function $u: {\mathbb {T}}^d \rightarrow {\mathbb {C}}$, we define

$$\begin{aligned} \Pi _0 u := \frac{1}{|{\mathbb {T}}^d_\Gamma |^{\frac{1}{2}}} \int _{{\mathbb {T}}^d} u(x)\, d\,x, \quad \Pi _0^\bot := \textrm{Id} - \Pi _0. \end{aligned}$$

Let ${\bar{g}}$ be a metric in the set of the admissible metrics ${{\mathcal {G}}}$ given in the definition 5.1. Exactly as in the case of the Beam equation, we consider a metric *g* of the form

5.26 $$\begin{aligned} g = \beta {\bar{g}}, \quad \beta \in {{\mathcal {B}}} := (\beta _1, \beta _2), \quad 0< \beta _1< \beta _2 < + \infty . \end{aligned}$$

we shall use the parameter $\beta$ in order to tune the resonances and to impose the non-resonance conditions required in order to apply Theorem 2.10. The precise statement of the long time existence for the QHD system is the following.

#### Theorem 5.13

Let ${\bar{g}} \in {{\mathcal {G}}}$. There exists a set of zero measure ${{\mathcal {B}}}^{(res)}\subset {{\mathcal {B}}}$, s.t. if $\beta \in {{\mathcal {B}}} {\setminus } {{\mathcal {B}}}^{(res)}$ and $g = \beta {\bar{g}}$, then, $\forall r\ge 2$ there exist $s_r$ and $\forall s>s_r$ $\exists \epsilon _{rs},c,C$ with the following property. For any initial datum $(\rho _0, \phi _0) \in H^s({\mathbb {T}}^d_\Gamma ) \times H^s({\mathbb {T}}^d_\Gamma )$ satisfying

$$\begin{aligned} \Vert \rho _0 \Vert _s + \Vert \Pi _0^\bot \phi _0 \Vert _s \le \epsilon \end{aligned}$$

there exists a unique solution $t \mapsto (\rho (t), \phi (t))$ of the system (QHD) satisfying the bound

$$\begin{aligned} \Vert \rho (t) \Vert _s + \Vert \Pi _0^\bot \phi (t) \Vert _s \le C \epsilon , \quad \forall \, |t| \le c\epsilon ^{-r}. \end{aligned}$$

Arguing as in the proof of Corollary 5.8, one can show

#### Corollary 5.14

Let $0< \beta _1 < \beta _2$. There exists a zero measure set ${{\mathcal {G}}}^{(res)}_{\beta _1, \beta _2} \subseteq {{\mathcal {G}}}_0(\beta _1, \beta _2)$ (where ${{\mathcal {G}}}_0(\beta _1, \beta _2)$ is defined in (5.13)) such that for any

$$\begin{aligned} g \in {{\mathcal {G}}}_0(\beta _1, \beta _2) \setminus {{\mathcal {G}}}^{(res)}_{\beta _1, \beta _2} \end{aligned}$$

the statements of theorem 5.13 hold.

The key tool in order to prove the latter almost global existence result 5.13 is to use a change of coordinates (the so called Madelung transformation) which allows to reduce the system (QHD) to a semilinear Schrödinger type equation. We shall implement this in the next sections.

#### Madelung transform

For $\lambda \in \mathbb {R}_{+}$, we define the change of variable (*Madelung transform*) 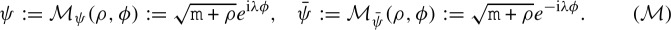 Notice that the inverse map has the form

5.27 $$\begin{aligned} \begin{aligned} \texttt{m}+\rho&=\mathcal {M}_\rho ^{-1}(\psi ,{\bar{\psi }}):=|\psi |^{2},\\ \phi&=\mathcal {M}_\phi ^{-1}(\psi ,{\bar{\psi }}):=\frac{1}{\lambda } \arctan \left( \frac{-\textrm{i} (\psi -\bar{\psi })}{\psi +\bar{\psi }}\right) . \end{aligned} \end{aligned}$$

In the following lemma we state a well-posedness result for the Madelung transform.

##### Lemma 5.15

Define $\kappa =(4\lambda ^{2})^{-1}$ and $\hbar :=\lambda ^{-1}=2\sqrt{\kappa }$. Then the following holds.

(*i*) Let $$s>\frac{d}{2}$$ and

$$\begin{aligned} \delta :=\frac{1}{\texttt{m}}\Vert \rho \Vert _{s}+\frac{1}{\sqrt{\kappa }}\Vert \Pi _0^{\bot }\phi \Vert _{s} , \qquad \sigma :=\Pi _0\phi . \end{aligned}$$

There is $C=C(s)>1$ such that, if $C(s)\delta \le 1$, then the function $\psi$ in ($\mathcal {M}$) satisfies

$$\begin{aligned} \Vert \psi -\sqrt{\texttt{m}}e^{\textrm{i} \lambda \sigma }\Vert _{s}\le 2\sqrt{\texttt{m}}\delta . \end{aligned}$$

(*ii*) Define

$$\begin{aligned} \delta ':=\inf _{\sigma \in {\mathbb {T}}}\Vert \psi -\sqrt{\texttt{m}}e^{\textrm{i} \sigma }\Vert _{s}. \end{aligned}$$

There is $C'=C'(s)>1$ such that, if $C'(s) \delta '(\sqrt{\texttt{m}})^{-1}\le 1$, then the functions $\rho ,$

$$\begin{aligned} \frac{1}{\texttt{m}}\Vert \rho \Vert _{s} +\frac{1}{\sqrt{\kappa }}\Vert \Pi _0^{\bot }\phi \Vert _{s}\le 8\frac{1}{\sqrt{\texttt{m}}}\delta '. \end{aligned}$$

##### Proof

see Lemma 2.1 in [33]. $\square$

We now rewrite equation (QHD) in the variable $(\psi ,\bar{\psi })$.

##### Lemma 5.16

Let $(\rho ,\phi )\in H^s_0({\mathbb {T}}^d)\times H^s({\mathbb {T}}^d)$ be a solution of (QHD) defined over a time interval [0, *T*], $T>0$, such that

$$\begin{aligned} \sup _{t\in [0,T)}\Big (\frac{1}{\texttt{m}}\Vert \rho (t,\cdot )\Vert _{s} +\frac{1}{\sqrt{\kappa }} \Vert \Pi _0^{\bot }\phi (t,\cdot )\Vert _{s} \Big )\le \epsilon \end{aligned}$$

for some $\epsilon >0$ small enough. Then the function $\psi$ defined in ($\mathcal {M}$) solves

5.28 $$\begin{aligned} {\left\{ \begin{array}{ll} \partial _{t}\psi =-\textrm{i} \big (-\frac{\hbar }{2}\Delta _g\psi +\frac{1}{\hbar }p(|\psi |^{2})\psi \big )\\ \psi (0)= \sqrt{\texttt{m}+\rho (0)}e^{\textrm{i} \phi (0)}. \end{array}\right. } \end{aligned}$$

##### Proof

See Lemma 2.2 in [33]. $\square$

Notice that the (5.28) is an Hamiltonian equation of the form

5.29 $$\begin{aligned} \partial _{t}\psi =-\textrm{i} \partial _{{\bar{\psi }}}\mathcal {H}(\psi ,\bar{\psi }),\qquad \mathcal {H}(\psi ,\bar{\psi })= \int _{\mathbb {T}^{d}} \big (\frac{\hbar }{2}|\nabla _g\psi |^{2}+\frac{1}{\hbar }P(|\psi |^{2})\big )dx, \end{aligned}$$

where $\partial _{\bar{\psi }}=(\partial _{\Re \psi }+\textrm{i} \partial _{\Im \psi })/2$. The Poisson bracket is defined by

5.30 $$\begin{aligned} \{\mathcal {H}, \mathcal {G}\}:=- \textrm{i} \int _{{\mathbb {T}}^d} \partial _{\psi }\mathcal {H}\partial _{\bar{\psi }}\mathcal {G} - \partial _{\bar{\psi }}\mathcal {H}\partial _{{\psi }}\mathcal {G} dx. \end{aligned}$$

#### Elimination of the zero mode

We introduce the set of variables

5.31 $$\begin{aligned} {\left\{ \begin{array}{ll} \psi _0= \alpha e^{-\textrm{i} \theta } &{} \alpha \in [0,+\infty ),\, \theta \in {\mathbb {T}}\\ \psi _j= z_j e^{-\textrm{i} \theta } &{} j\in {\mathbb {Z}}^d \setminus \{0\}, \end{array}\right. } \end{aligned}$$

which are the polar coordinates for $j=0$ and a phase translation for $j\not =0$. Rewriting (5.29) in Fourier coordinates one has

$$\begin{aligned} \textrm{i} \partial _t \psi _j = \partial _{\bar{\psi _j}}\mathcal {H} (\psi ,{\bar{\psi }}), \quad j\in {\mathbb {Z}}^d, \end{aligned}$$

where $\mathcal {H}$ is defined in (5.29). We define also the zero mean variable

5.32 $$\begin{aligned} z:= \sum _{j\in {\mathbb {Z}}^d \setminus \{ 0\} } z_j e^{\textrm{i} j\cdot x}. \end{aligned}$$

By (5.31) and (5.32) one has

5.33 $$\begin{aligned} \psi = ( \alpha + z) e^{\textrm{i} \theta }, \end{aligned}$$

and it is easy to prove that the quantity

$$\begin{aligned} \texttt{m}:= \sum _{j\in {\mathbb {Z}}^d} | \psi _j|^2= \alpha ^2 + \sum _{j \in {\mathbb {Z}}^d \setminus \{ 0 \}} | z_j|^2 \end{aligned}$$

is a constant of motion for (5.28). Using (5.31), one can completely recover the variable $\alpha$ in terms of $\{ z_j\}_{j\in {\mathbb {Z}}^d {\setminus } \{0\}}$ as

$$\begin{aligned} \alpha = \sqrt{ \texttt{m}- \sum _{j \in {\mathbb {Z}}^d \setminus \{ 0 \}} | z_j|^2}. \end{aligned}$$

Note also that the $(\rho ,\phi )$ variables in (5.27) do not depend on the angular variable $\theta$ defined above. This implies that system (QHD) is completely described by the complex variable *z*. On the other hand, using

$$\begin{aligned} \partial _{\bar{\psi _j}}\mathcal {H}(\psi e^{\textrm{i} \theta },\bar{\psi e^{\textrm{i} \theta }}) = \partial _{\bar{\psi _j}}\mathcal {H}(\psi ,{\bar{\psi }})e^{\textrm{i} \theta }, \end{aligned}$$

one obtains

5.34 $$\begin{aligned} {\left\{ \begin{array}{ll} \textrm{i} \partial _t \alpha +\partial _t\theta \alpha = \Pi _0\left( p( |\alpha +z|^2) (\alpha + z) \right) \\ \textrm{i} \partial _t z_j + \partial _t\theta z_j= \frac{\partial \mathcal {H}}{\partial {\bar{\psi }}_j}(\alpha + z ,\alpha +{\bar{z}}). \end{array}\right. } \end{aligned}$$

Taking the real part of the first equation in (5.34) we obtain

5.35 $$\begin{aligned} \partial _t\theta = \frac{1}{\alpha } \Pi _0\left( \frac{1}{\hbar }p( |\alpha +z|^2)\Re (\alpha + z) \right) = \frac{1}{2\alpha } \partial _{\bar{\alpha }}\mathcal {H}(\alpha , z, {\bar{z}}), \end{aligned}$$

where

$$\begin{aligned} \tilde{\mathcal {H}}(\alpha , z, {\bar{z}}):= \frac{\hbar }{2}\int _{{\mathbb {T}}^d} (- \Delta _g)z\cdot \bar{z}\textrm{d}x + \frac{1}{\hbar }\int _{{\mathbb {T}}^d} G(| \alpha +z|^2)\, \textrm{d}x. \end{aligned}$$

By (5.35), (5.34) and using that

$$\begin{aligned} \partial _{\bar{\psi _j}}\mathcal {H}(\alpha + z,\alpha +{\bar{z}}) = \partial _{\bar{z_{j}}}\tilde{\mathcal {H}}(\alpha , z, {\bar{z}}), \end{aligned}$$

one obtains

5.36 $$\begin{aligned} \begin{aligned} \textrm{i} \partial _t z_j =&\partial _{\bar{z_j}}\tilde{\mathcal {H}}(\alpha , z, {\bar{z}}) - \frac{z_j}{2\alpha } \partial _{\alpha }\tilde{\mathcal {H}}(\alpha , z, {\bar{z}})= \partial _{\bar{z_j}}\mathcal {K}_{\texttt{m}}( z, {\bar{z}}), \quad j\not =0, \end{aligned} \end{aligned}$$

where

5.37 $$\begin{aligned} \mathcal {K}_{\texttt{m}}(z,{\bar{z}}):= \tilde{\mathcal {H}}(\alpha ,z,{\bar{z}})_{|\alpha =\sqrt{\texttt{m}- \sum _{j\not =0} | z_j|^2}}. \end{aligned}$$

We resume the above discussion in the following lemma.

##### Lemma 5.17

The following holds.

(*i*) Let $$s>\frac{d}{2}$$ and

$$\begin{aligned} \delta :=\frac{1}{\texttt{m}}\Vert \rho \Vert _{s}+\frac{1}{\sqrt{\kappa }}\Vert \Pi _0^{\bot }\phi \Vert _{s} , \quad \theta :=\Pi _0\phi . \end{aligned}$$

There is $C=C(s)>1$ such that, if $C(s)\delta \le 1$, then the function *z* in (5.32) satisfies

$$\begin{aligned} \Vert z\Vert _{s}\le 2\sqrt{\texttt{m}}\delta . \end{aligned}$$

(*ii*) Define

$$\begin{aligned} \delta ':=\Vert z\Vert _{s}. \end{aligned}$$

There is $C'=C'(s)>1$ such that, if $C'(s) \delta '(\sqrt{\texttt{m}})^{-1}\le 1$, then the functions $\rho ,$

$$\begin{aligned} \frac{1}{\texttt{m}}\Vert \rho \Vert _{s}+\frac{1}{\sqrt{\kappa }}\Vert \Pi _0^{\bot }\phi \Vert _{s} \le 16\frac{1}{\sqrt{\texttt{m}}}\delta '. \end{aligned}$$

(*iii*) Let $(\rho ,\phi )\in H^s_0({\mathbb {T}}^d)\times H^s({\mathbb {T}}^d)$ be a solution of (QHD) defined over a time interval [0, *T*], $T>0$, such that

$$\begin{aligned} \sup _{t\in [0,T)}\Big (\frac{1}{\texttt{m}}\Vert \rho (t,\cdot )\Vert _{s} +\frac{1}{\sqrt{\kappa }} \Vert \Pi _0^{\bot }\phi (t,\cdot )\Vert _{s} \Big )\le \epsilon \end{aligned}$$

for some $\epsilon >0$ small enough. Then the function $z\in H^s_0({\mathbb {T}}^d)$ defined in (5.32) solves (5.36).

##### Proof

See Lemma 2.4 in [33]. $\square$

##### Remark 5.18

Using (5.27) and (5.33) one can study the system (QHD) near the equilibrium point $(\rho ,\phi )=(0,0)$ by studying the complex hamiltonian system

5.38 $$\begin{aligned} \textrm{i} \partial _t z = \partial _{\bar{z}}\mathcal {K}_{\texttt{m}}(z,\bar{z}) \end{aligned}$$

near the equilibrium $z=0$, where $\mathcal {K}_{\texttt{m}}(z,\bar{z})$ is the Hamiltonian in (5.37). Note also that the natural phase-space for (5.38) is the complex Sobolev space $H_0^s({\mathbb {T}}^d)$, $s\in {\mathbb {R}}$, of complex Sobolev functions with zero mean.

By Lemma 5.17, one has that Theorem 5.13 will be deduced by the following Proposition

##### Proposition 5.19

Let ${\bar{g}} \in {{\mathcal {G}}}$. There exists a set of zero measure ${{\mathcal {B}}}^{(res)}\subset {{\mathcal {B}}}$, s.t. if $\beta \in {{\mathcal {B}}} {\setminus } {{\mathcal {B}}}^{(res)}$ and $g = \beta {\bar{g}}$ then, $\forall r\ge 2$ there exist $s_r$ and $\forall s>s_r$ $\exists \epsilon _{rs},c,C$ with the following property. For any initial datum $z_0 \in H^s_0({\mathbb {T}}^d)$ satisfying

$$\begin{aligned} \Vert z_0 \Vert _s \le \epsilon \end{aligned}$$

there exists a unique solution $t \mapsto z(t)$ of the equation (5.36) satisfying the bound

$$\begin{aligned} \Vert z(t) \Vert _s \le C \epsilon , \qquad \forall \, |t| \le c\epsilon ^{-r}. \end{aligned}$$

The rest of this section is dedicated to the proof of the latter Proposition.

#### Taylor expansion of the Hamiltonian

In this section we shall use the notations introduced in Sects. 2.1, 2.2. The only difference is that, since we shall restrict to the space of zero average functions, in all the definitions given in Sects. 2.1, 2.2, one has to replace ${\mathbb {Z}}^d$ by ${\mathbb {Z}}^d {\setminus } \{ 0 \}$ and ${{\mathcal {Z}}}^d$ by ${{\mathcal {Z}}}^d_0:= ({\mathbb {Z}}^d {\setminus } \{ 0 \}) \times \{ +, - \}$. In order to study the stability of $z=0$ for (5.38) it is useful to expand $\mathcal {K}_{\texttt{m}}$ at $z=0$. We have

5.39 $$\begin{aligned} \mathcal {K}_{\texttt{m}}(z,{\bar{z}})&= \frac{\hbar }{2} \int _{{\mathbb {T}}^d} (- \Delta _g)z\cdot \bar{z}\, \textrm{d} x + \frac{1}{\hbar } \int _{{\mathbb {T}}^d} P\Big (\big | \sqrt{\texttt{m}- \sum _{j\not =0} | z_j|^2}+ z\big |^2\Big )\, \textrm{d}x \nonumber \\&=(2\pi )^d \frac{P(\texttt{m})}{\hbar } +\mathcal {K}_{\texttt{m}}^{(2)}(z,{\bar{z}}) + \sum _{r= 3}^{N-1} \mathcal {K}_{\texttt{m}}^{(r)}(z,{\bar{z}})+R^{(N)}(z,{\bar{z}}), \end{aligned}$$

where

$$\begin{aligned} \mathcal {K}_{\texttt{m}}^{(2)}(z,{\bar{z}})= \frac{1}{2} \int _{{\mathbb {T}}^d} \frac{\hbar }{2} (- \Delta _g) z\cdot \bar{z}\, \textrm{d} x + \frac{p'(\texttt{m})\texttt{m}}{\hbar }\int _{{\mathbb {T}}^d} \frac{1}{2}( z+{\bar{z}})^2\, \textrm{d}x, \end{aligned}$$

for any $r=3, \cdots , N-1$, $\mathcal {K}_{\textrm{m}}^{(r)}(z,{\bar{z}})$ is an homogeneous multilinear Hamiltonian function of degree *r* of the form

$$\begin{aligned} \mathcal {K}_{\texttt{m}}^{(r)}(z,{\bar{z}})= \sum _{\begin{array}{c} \sigma \in \{-1,1\}^r,\ j\in ({\mathbb {Z}}^d\setminus \{0\})^r\\ \sum _{i=1}^r\sigma _i j_i=0 \end{array}} (\mathcal {K}_{\texttt{m}}^{(r)})_{\sigma ,j} z_{j_1}^{\sigma _1}\cdots z_{j_r}^{\sigma _r}, \qquad |(\mathcal {K}_{\texttt{m}}^{(r)})_{\sigma ,j}|\lesssim _{r}1, \end{aligned}$$

and

$$\begin{aligned} \Vert X_{R^{(N)}}(z)\Vert _{s}\lesssim _s \Vert z\Vert _{H^s}^{N-1}, \qquad \forall \, z\in B_{1}( H_0^{s}(\mathbb {T}^{d}) . \end{aligned}$$

This implies that ${{\mathcal {K}}}_m^{(r)}$ is in the class ${{\mathcal {P}}}_r$. The vector field of the Hamiltonian in (5.39) has the form

$$\begin{aligned} \begin{aligned} \partial _t\begin{bmatrix} z \\ {\bar{z}}\end{bmatrix}= \begin{bmatrix}-\textrm{i} \partial _{\bar{z}} \mathcal {K}_{\texttt{m}}\\ \textrm{i} \partial _{ z} \mathcal {K}_{\texttt{m}}\end{bmatrix}&=-\textrm{i} \begin{pmatrix} \frac{ \hbar \Delta _g}{2} + \frac{\texttt{m} p'(\texttt{m})}{\hbar }&{} \frac{\texttt{m}p'(\texttt{m})}{\hbar }\\ -\frac{\texttt{m} p'(\texttt{m})}{\hbar } &{}\frac{\hbar \Delta _g}{2} - \frac{\texttt{m} p'(\texttt{m})}{\hbar }\end{pmatrix} \begin{bmatrix} z \\ {\bar{z}} \end{bmatrix}\\&\quad + \sum _{r=3}^{N-1}\begin{bmatrix} -\textrm{i} \partial _{\bar{z}} \mathcal {K}_{\texttt{m}}^{(r)}\\ \textrm{i} \partial _{ z} \mathcal {K}_{\texttt{m}}^{(r)}\end{bmatrix}+ \begin{bmatrix} -\textrm{i} \partial _{\bar{z}} R^{(N)}\\ \textrm{i} \partial _{ z} R^{(N)}\end{bmatrix}. \end{aligned} \end{aligned}$$

Let us now introduce the $2\times 2$ matrix of operators

$$\begin{aligned} \mathcal {C}:=\frac{1}{\sqrt{2\omega (D) A(D,\texttt{m})}} \left( \begin{matrix} A(D,\texttt{m}) &{} -\tfrac{1}{2}\texttt{m}p'(\texttt{m})\\ -\tfrac{1}{2}\texttt{m}p'(\texttt{m}) &{} A(D,\texttt{m}) \end{matrix} \right) , \end{aligned}$$

with

$$\begin{aligned} A(D,\texttt{m}):= \omega (D) +\tfrac{\hbar }{2} (- \Delta _g) +\tfrac{1}{2}\texttt{m}p'(\texttt{m}), \end{aligned}$$

and where $\omega (D)$ is the Fourier multiplier with symbol

5.40 $$\begin{aligned} \begin{aligned}&\sqrt{ \frac{\hbar ^2}{4} |j|_{g}^4+ \texttt{m}p'(\texttt{m}) |j|_{g}^2} = \frac{\hbar }{2}\omega _j , \quad j \in {\mathbb {Z}}^d \setminus \{ 0 \} \\&\omega _j :=\sqrt{|j_g|^4 + \delta |j_g|^2}, \qquad \delta := \frac{4\texttt{m}p'(\texttt{m})}{\hbar ^2}. \end{aligned} \end{aligned}$$

Notice that, by using (5.25), the matrix $\mathcal {C}$ is bounded, invertible and symplectic, with estimates

$$\begin{aligned} \Vert \mathcal {C}^{\pm 1}\Vert _{\mathcal {L}{(H^s_0\times H^s_0,\,\, H^s_0\times H^s_0)}}\le 1+\sqrt{k}\beta ,\quad \beta :=\frac{\texttt{m}p'(\texttt{m})}{k}. \end{aligned}$$

Consider the change of variables

$$\begin{aligned} \begin{bmatrix} w \\ {\bar{w}} \end{bmatrix} := \mathcal {C}^{-1} \begin{bmatrix} z \\ {\bar{z}} \end{bmatrix}. \end{aligned}$$

then the Hamiltonian (5.39) reads

$$\begin{aligned} \begin{aligned}&\widetilde{\mathcal {K}}_{\texttt{m}}= \widetilde{\mathcal {K}}^{(2)}_{\texttt{m}}+ \sum _{k = 3}^{N - 1} \widetilde{{\mathcal {K}}}_m^{(r)} + \tilde{R}_N\\&\widetilde{\mathcal {K}}^{(2)}_{\texttt{m}}(w,\bar{w}):= \mathcal {K}^{(2)}_{\texttt{m}}\Big (\mathcal {C} \begin{bmatrix} w \\ {\bar{w}} \end{bmatrix}\Big ):=\frac{1}{2} \int _{\mathbb {T}^{d}}\omega (D)w\cdot \bar{w}\textrm{d}x,\\&\widetilde{\mathcal {K}}^{(i)}_{\texttt{m}} \in {{\mathcal {P}}}_i \quad i = 3, \ldots , N - 1, \\&\Vert X_{{\widetilde{R}}_N}(w) \Vert _s \lesssim _s \Vert w \Vert _s^{N - 1}, \quad \forall \Vert w \Vert _s \ll 1. \end{aligned} \end{aligned}$$

From the latter properties, one deduces that the perturbation

$$\begin{aligned} P= \sum _{k = 3}^{N - 1} \widetilde{{\mathcal {K}}}_m^{(r)} + \tilde{R}_N, \end{aligned}$$

is in the class ${\mathcal {P}}$ of Definition 2.4.

The verification of (F.3) goes exactly as in the case of the Schrödinger equation, since also in this case $\omega _j = |j|_g^2 + O(1)$. The asymptotic condition (F.1) is also trivially fulfilled with $\beta =2$. The main point is to verify the nonresonance conditions (F.2) and (NR.1), (NR.2). This will be done in the next subsection.

#### Non-resonance conditions for (QHD)

According to the Sect. 5.2 on the Beam equation, we fix the metric ${\bar{g}} \in {{\mathcal {G}}}$ and we consider $g = \beta \, {\bar{g}}$, $\beta _1 \le \beta \le \beta _2$. We shall verify the non-resonance conditions on the frequencies $\omega _j$ in (5.40). By the property (5.20),

5.41 $$\begin{aligned} \begin{aligned} \omega _j&= \sqrt{|j|_g^4 + \delta |j|_g^2} = \sqrt{\beta ^4 |j|_{{\bar{g}}}^4 + \beta ^2 \delta |j|_{{\bar{g}}}^2} = \beta ^2 \Omega _j, \\ \Omega _j&:= |j|_{{\bar{g}}} \sqrt{|j|_{{\bar{g}}}^2 + \frac{\delta }{\beta ^2}}, \quad j \in {\mathbb {Z}}^d \setminus \{ 0 \}. \end{aligned} \end{aligned}$$

Since $$\beta _2 \ge \beta \ge \beta _1 > 0$$,

$$\begin{aligned} \Big |\sum _{i = 1}^r \sigma _i \omega _{j_i} \Big | = \beta ^2 \Big | \sum _{i = 1}^r \sigma _i \Omega _{j_i}\Big | \ge \beta _1^2 \Big | \sum _{i = 1}^r \sigma _i \Omega _{j_i}\Big | \end{aligned}$$

one can verify non resonance conditions on $\Omega _j$. Since the map

5.42 $$\begin{aligned} (\beta _1, \beta _2) \rightarrow (\zeta _1, \zeta _2) := \Big (\frac{\delta }{\beta _2^2}, \frac{\delta }{\beta _1^2} \Big ), \qquad \beta \mapsto \zeta := \frac{\delta }{\beta ^2} \end{aligned}$$

is an analytic diffeomorphism, we can introduce $\zeta = \delta / \beta ^2$ as parameter in order to tune the resonances. Hence we verify non resonance conditions on the frequencies

5.43 $$\begin{aligned} \Omega _j \equiv \Omega _j (\zeta ) =|j|_{{\bar{g}}} \sqrt{|j|_{{\bar{g}}}^2 + \zeta }, \quad j \in {\mathbb {Z}}^d \setminus \{ 0 \}. \end{aligned}$$

##### Lemma 5.20

Assume that the metric ${\bar{g}} \in {{\mathcal {G}} }$. For any $K\le N$, consider *K* indexes ${j_{1}},...,{j_{K}}$ with $\left| {j_{1}}\right| _g< \ldots < \left| {j_{K}}\right| _g\le N$; and consider the determinant

$$\begin{aligned} D:=\left| \begin{matrix} \Omega _{{j_{1}}}&{} \Omega _{{j_{2}}}&{}.&{} .&{}.&{}\Omega _{{j_{K}}} \\ \partial _\zeta \Omega _{j_1} &{} \partial _\zeta \Omega _{j_2}&{} .&{} .&{}.&{} \partial _\zeta \Omega _{j_K} \\ .&{} .&{} .&{} .&{} .&{}. \\ .&{} .&{} .&{} .&{} .&{}. \\ \partial _\zeta ^{K - 1} \Omega _{j_1}&{} \partial _\zeta ^{K - 1} \Omega _{j_2}&{} .&{} .&{}.&{} \partial _\zeta ^{K - 1} \Omega _{j_K} \end{matrix} \right| \end{aligned}$$

One has

$$\begin{aligned} D \ge \frac{C}{N^{\eta K^2}} \end{aligned}$$

for some constant $\eta \equiv \eta _d > 0$ large enough, depending only on the dimension *d*.

##### Proof

The dispersion relation is slightly different w.r. to the one of the Beam equation, hence in this proof we just highlight the small differences w.r. to Lemma 5.11. For any $i = 1, \ldots , K$, for any $n = 0, \ldots , K - 1$, one computes

$$\begin{aligned} \partial _\zeta ^n \Omega _{j_i}(\zeta ) = C_n |j_i|_{{\bar{g}}} (|j_i|_{{\bar{g}}}^2 + \zeta )^{\frac{1}{2} - n} \end{aligned}$$

for some constant $C_n \ne 0$. This implies that

$$\begin{aligned} D \ge C \prod _{i = 1}^K \Big (|j_i|_{{\bar{g}}} \sqrt{|j_i|_{{\bar{g}}}^2 + \zeta } \Big ) |\textrm{det}(A)| \end{aligned}$$

where the matrix *A* is defined as

$$\begin{aligned} A = \begin{pmatrix} 1&{} 1 &{}.&{}.&{}.&{}1 \\ x_1&{}x_2&{}.&{}.&{}.&{}x_K \\ .&{}.&{}.&{}.&{}.&{}. \\ .&{}.&{}.&{}.&{}.&{}. \\ x_1^{K - 1}&{} x_2^{K - 1}&{}.&{}.&{}.&{}x_K^{K - 1} \end{pmatrix} \end{aligned}$$

where

$$\begin{aligned} x_i:= \frac{1}{|j_i|_{{\bar{g}}}^2 + \zeta }, \quad i = 1, \ldots , K. \end{aligned}$$

This is a Van der Monde determinant. Thus we have

$$\begin{aligned} \begin{aligned} |\textrm{det}(A)|&= \prod _{1 \le i< \ell \le K} |x_i - x_\ell | = \prod _{1 \le i< \ell \le K} \Big | \frac{1}{|j_i|_{{\bar{g}}}^2 + \zeta } - \frac{1}{|j_\ell |_{{\bar{g}}}^2 + \zeta } \Big | \\&\ge \prod _{1 \le i < \ell \le K} \frac{||j_i|_{{\bar{g}}}^2 - |j_\ell |_{{\bar{g}}}^2|}{||j_i|_{{\bar{g}}}^2 + \zeta | ||j_\ell |_{{\bar{g}}}^2 + \zeta |} {\mathop {\ge }\limits ^{Lemma \,\,5.10}} C N^{- K^2(\tau _* + 4)} \end{aligned} \end{aligned}$$

which implies the thesis. $\square$

Exploiting this Lemma, and following step by step the proof of Lemma 12 of [2] one gets

##### Lemma 5.21

Let ${\bar{g}} \in {{\mathcal {G}}}$. Then for any *r* there exists $\tau _r$ with the following property: for any positive $\gamma$ small enough there exists a set $I_\gamma \subset (\zeta _1, \zeta _2)$ such that $\forall \zeta \in I_\gamma$ one has that for any $N\ge 1$ and any set $J_1,...,J_r$ with $|J_l|\le N$ $\forall l$, one has

$$\begin{aligned} \sum _{l=1}^r\sigma _l\Omega _{j_l}\not =0\qquad \Longrightarrow \qquad \left| \sum _{l=1}^r\sigma _l\Omega _{j_l}\right| \ge \frac{\gamma }{N^{\tau }}\ . \end{aligned}$$

Moreover one has

$$\begin{aligned} \left| [\zeta _1,\zeta _2]\setminus I_\gamma \right| \le C \gamma ^{1/r}\ . \end{aligned}$$

By recalling the diffeomorphism (5.42), one has that the set

$$\begin{aligned} {{\mathcal {I}}}_\gamma := \{ \beta \in [\beta _1, \beta _2]: \delta /\beta ^2 \in I_\gamma \} \end{aligned}$$

satisfies the estimate

$$\begin{aligned} |(\beta _1, \beta _2) \setminus {{\mathcal {I}}}_\gamma | \lesssim \gamma ^{\frac{1}{r}} \end{aligned}$$

Now, if we take $\beta \in {{\mathcal {I}}}_\gamma$ and if $\sum _{i=1}^r\sigma _i\omega _{j_i}\not =0$, one has that (recall (5.41))

$$\begin{aligned} \begin{aligned} \Big |\sum _{i = 1}^r \sigma _i \omega _{j_i} \Big |&= \beta ^2 \Big | \sum _{i = 1}^r \sigma _i \Omega _{j_i}\Big | {\mathop {\ge }\limits ^{\beta _1 \le \beta \le \beta _2}}\beta _1^2 \Big | \sum _{i = 1}^r \sigma _i \Omega _{j_i}\Big | \\&\ge \frac{\beta _1^2 \gamma }{N^\tau }. \end{aligned} \end{aligned}$$

By the above result, one has that, if

$$\begin{aligned} \beta \in \bigcup _{\gamma >0}{{\mathcal {I}}}_\gamma \, \end{aligned}$$

then (NR.2) holds and furthermore $\bigcup _{\gamma >0}{{\mathcal {I}}}_\gamma$ has full measure. Hence the claimed statement follows by defining ${{\mathcal {B}}}^{(res)}:= {{\mathcal {B}}} {\setminus } \Big ( \bigcup _{\gamma >0}{{\mathcal {I}}}_\gamma \Big )$.

### Stability of Plane Waves in NLS

Consider the NLS

5.44 $$\begin{aligned} \textrm{i}\psi _t=-\Delta _g \psi +f(|\psi |^2)\psi , \end{aligned}$$

with $f\in C^\infty ({\mathbb {R}},{\mathbb {R}})$, $f(0) = 0$ and $g = \beta {\bar{g}}$, ${\bar{g}}\in {\mathcal {G}}$ and $\beta \in (\beta _1, \beta _2) \subset (0, + \infty )$. (recall the Definition 5.1). The equation (5.44) admits solutions of the form

5.45 $$\begin{aligned} \psi _{*,m}(x,t)=ae^{\textrm{i}(m\cdot x-\nu t)},\quad m\in {\mathbb {Z}}^d \end{aligned}$$

with $\nu =|m|_g^2+f(a^2)$ and $a > 0$. In order to state the next stability theorem, we need that a suitable condition between $f'(a^2)$ and the metric *g* is satisfied. For this reason, we slightly modify the definition of ${{\mathcal {G}}}_0$ in 5.1. We then re-define ${{\mathcal {G}}}_0$ in the following way: fix $K > 0$, we define

5.46 $$\begin{aligned} \mathcal {G}_{0}:=\left\{ \left( g_{ij}\right) _{i\le j}\in {\mathbb {R}}^{\frac{d (d + 1)}{2}}\; : \; \inf _{x \ne 0} \frac{g(x, x)}{|x|^2} > K \right\} \end{aligned}$$

The definition of the admissible set ${{\mathcal {G}}}$ is then the same in which one replace this new set ${{\mathcal {G}}}_0$ with its hold definition. The main theorem of this section is the following.

#### Theorem 5.22

Assume that $$0< \beta _1 < \beta _2$$, $${\bar{g}} \in {{\mathcal {G}}}$$, $$2f'(a^2)< \beta _1^2 K^2$$, $$f'(a^2) \ne 0$$ (where $$K > 0$$ is the constant appearing in (5.46)). Then there exists a set of zero measure $${{\mathcal {B}}}^{(res)} \subset {{\mathcal {B}}}:= (\beta _1, \beta _2)$$, such that for $$\beta \in {{\mathcal {B}}} {\setminus } {{\mathcal {B}}}^{(res)} $$ for $$g = \beta {\bar{g}}$$, then, for any $$ r\ge 3$$, there exist $$s_r>0$$ such that the following holds. For any $$ s>s_r$$ and any $$ m\in {\mathbb {Z}}^d$$ there exist constants $$ \epsilon _{rsm},c,C$$ such that if the initial datum $$\psi _0$$ for (5.44) fulfills

5.47 $$\begin{aligned} \left\| \psi _0\right\| _{L^2}=a\sqrt{|{\mathbb {T}}^d|_g}, \quad \epsilon :=\left\| \psi _0-\psi _{*,m}(.,0) \right\| _{H^s}<\epsilon _{srm}, \end{aligned}$$

then the corresponding solution fulfills

5.48 $$\begin{aligned} \left\| \psi (t)-\psi _{*,m}(.,t)\right\| _{s}\le C\epsilon ,\quad \forall \; \left| t\right| \le c\epsilon ^{-r}. \end{aligned}$$

Arguing as in the proof of Corollary 5.8, one can show also in this case the following

#### Corollary 5.23

Let $0< \beta _1 < \beta _2$. There exists a zero measure set ${{\mathcal {G}}}^{(res)}_{\beta _1, \beta _2} \subseteq {{\mathcal {G}}}_0(\beta _1, \beta _2)$, where ${{\mathcal {G}}}_0(\beta _1, \beta _2):= \big \{ g \in {{\mathcal {G}}}_0: \beta _1 \le \Vert g \Vert _2 \le \beta _2 \big \}$, such that for any $g \in {{\mathcal {G}}}_0(\beta _1, \beta _2) {\setminus } {{\mathcal {G}}}^{(res)}_{\beta _1, \beta _2}$ the statements of theorem 5.22 hold.

The rest of this subsection is devoted to sketch the proof of Theorem 5.22, which follows exactly the proof of the corresponding theorem in [29] except that in the case of nonresonant tori one has to substitute the nonresonant condition by [29] with our nonresonance and structure conditions (see Hypotheses 2.5, 2.8).

We start by reducing the problem to a problem of stability of the origin of a system of the form (2.14).

First it is easy to see that introducing the new variables $\varphi$ by

$$\begin{aligned} \varphi (x,t)=e^{-\textrm{i}m\cdot x}e^{-\textrm{i}t\left| m\right| ^2}\psi (x+2mt,t), \end{aligned}$$

then $\varphi$ still fulfills (5.44), but $\psi _{*,m}(x,t)$ is changed to $a e^{-\textrm{i}\nu t}$ with $\nu = f(a^2)$.

The idea of [29] is to exploit that $\varphi (x)=a$ appears as an elliptic equilibrium of the reduced Hamiltonian system obtained applying Marsden Weinstein procedure to (5.44) in order to reduce the Gauge symmetry. We recall that according to Marsden Weinstein procedure (following [29]), when one has a system invariant under a one parameter symmetry group, then there exists an integral of motion (the $L^2$ norm in this case), and the effective dynamics occurs in the quotient of the level surface of the integral of motion with respect to the group action. This is the same procedure exploited in Sect. 5.3 for the QHD system. The effective system has a Hamiltonian which is obtained by restricting the Hamiltonian to the level surface. Such a Hamiltonian is invariant under the symmetry group associated to the integral of motion.

More precisely, consider the zero mean variable

$$\begin{aligned} z(x):=\frac{1}{|{\mathbb {T}}^d|_g^{1/2}}\sum _{j\in {\mathbb {Z}}^d\setminus \left\{ 0\right\} } z_j e^{\textrm{i}j\cdot x}\, \end{aligned}$$

and the substitution

5.49 $$\begin{aligned} \varphi (x)=e^{\textrm{i}\theta } (\sqrt{a^2-\left| {\mathbb {T}}^d\right| _g\left\| z\right\| ^2_{L^2}}+z(x) ) \end{aligned}$$

where $\theta \in {\mathbb {T}}$ is a parameter along the orbit of the Gauge group, Notice that $\varphi$ belongs to the level surface $\Vert \varphi \Vert _{L^2}=a\sqrt{\left| {\mathbb {T}}^d\right| _g}$ and *z*(*x*) is the new free variable. In this case it also turns out that this is a canonical variable (as it can be verified by the theory of [3]). Thus the Hamiltonian for the reduced system turns out to be

$$\begin{aligned} H_a(z,{\bar{z}})=\int _{{\mathbb {T}}^d} \left( {\bar{\varphi }}(-\Delta \varphi )+F(|\varphi |^2)\right) dx , \end{aligned}$$

with $\varphi$ given by (5.49). The explicit form of the Hamiltonian and its expansion were computed in [29] who showed that all the terms of the Taylor expansion of $H_a$ have zero momentum and that all the nonlinear terms are bounded, so, with our language, the nonlinear part is of class ${\mathcal {P}}$. Considering the quadratic part, [29] showed that there exists a linear transformation preserving $H^s$ norms and the zero momentum condition, such that the quadratic part takes the form (2.15) with

5.50 $$\begin{aligned} \omega _j=\sqrt{|j|_g^4-f'(a^2)|j|_g^2}\ . \end{aligned}$$

The system is now suitable for the application of Theorem 2.10. We do not give the details, since the verification of the nonresonance and structural assumptions are done exactly in the same way as in the previous cases. Indeed one can prove the nonresonance conditions on the frequencies (5.50) reasoning as done in Sect. 5.3.4.

## A Technical Lemma

**In this section by** $\ell ^2_s$ **we mean** $\ell ^2_s({\mathbb {Z}}^d;{\mathbb {C}})$ **.**

### Lemma A.1

Let

$$\begin{aligned} X:\underbrace{\ell ^2_s\times ...\times \ell ^2_s}_{r-\text {times}}\rightarrow \ell ^2_s, \end{aligned}$$

be a symmetric *r*-linear $X(u^{(1)},...,u^{(r)})=\left( X_j(u^{(1)},...,u^{(r)})\right) _{j\in {\mathbb {Z}}^d}$ with the property that there exist $\sigma _0,\sigma _1,...,\sigma _r$, with $\sigma _l\in \left\{ -1,1\right\}$ such that

A.1 $$\begin{aligned} X_j(u^{(1)},...,u^{(r)})=\sum _{{\begin{array}{c}j_1,...,j_r\in {\mathbb {Z}}^d \\ \sigma _0j+\sum _{l=1}^{r}\sigma _lj_l=0 \end{array}} }X_{j,j_1,...,j_r} u_{j_1}^{(1)}....u_{j_r}^{(r)}, \end{aligned}$$

and $X_{j,j_1,...,j_r}$ completely symmetric with respect to any permutation of the indexes $j,j_1,...,j_r$ fulfilling

$$\begin{aligned} \sup _{j,j_1,...,j_r\in {\mathbb {Z}}^{d}}\left| X_{ j,j_1,...,j_r}\right| <\infty . \end{aligned}$$

Then, for any $s>s_0>d/2$ there exists a constant $C_{s,r}>0$ such that one has

$$\begin{aligned} \begin{aligned} \left\| X(u^{(1)},...,u^{(r)})\right\| _s&\le C_{s,r} \sup _{j,j_1,...,j_r\in {\mathbb {Z}}^d}\left| X_{ j,j_1,...,j_r}\right| \times \\&\quad \times \sum _{l=1}^{r} \Vert u^{(1)} \Vert _{s_0}....\Vert u^{(l-1)} \Vert _{s_0} \Vert u^{(l)} \Vert _{s} \Vert u^{(l+1)} \Vert _{s_0}...\Vert u^{(r)} \Vert _{s_0}\ . \end{aligned} \end{aligned}$$

### Proof

One has

$$\begin{aligned} \left\| X(u^{(1)},...,u^{(r)})\right\| _s&= \sum _{j}\langle j\rangle ^{2s} \left| \sum _{{\begin{array}{c}j_1,...,j_r\in {\mathbb {Z}}^d \\ \sigma _0j+\sum _{l=1}^{r}\sigma _lj_l=0 \end{array}}} X_{j,j_1,...,j_r} u_{j_1}^{(1)}\cdots u_{j_r}^{(r)} \right| ^{2}\\&\le \sup _{j,j_1,...,j_r} \left| X_{ j,j_1,...,j_r}\right| ^2 \sum _{j}\langle j\rangle ^{2s} \left( \sum _{{\begin{array}{c}j_1,\ldots ,j_r\in {\mathbb {Z}}^d \\ \sigma _0j+\sum _{l=1}^{r}\sigma _lj_l=0 \end{array}} } |u_{j_1}^{(1)}\cdots u_{j_r}^{(r)} | \right) ^{2} \end{aligned}$$

To fix ideas consider first the case $\sigma _l=1$ $\forall l$, then the bracket is the *j*-th Fourier coefficient of the function $v(x)=u^{(1)}(x)\cdots u^{(r)}(x)$, with

$$\begin{aligned} u^{(l)}(x)=\sum _{j\in {\mathbb {Z}}^d}\frac{1}{|{\mathbb {T}}^d|^{1/2}} u^{(l)}_{j}e^{\textrm{i}j\cdot x}\, \end{aligned}$$

for which it is well known that

$$\begin{aligned} \left\| v\right\| _s \le C_{s,r} \sum _{l=1}^{r} \Vert u^{(1)} \Vert _{s_0}....\Vert u^{(l-1)} \Vert _{s_0} \Vert u^{(l)} \Vert _{s} \Vert u^{(l+1)} \Vert _{s_0}...\Vert u^{(r)} \Vert _{s_0}\ . \end{aligned}$$

then the thesis immediately follows. To deal with the case of different signs every time one has $$\sigma _l=-1$$ one simply substitutes $$\overline{u^{(l)}}$$ to $$u^{(l)} $$. This concludes the proof. $$\square $$

## Footnotes

1 Recall conditions *F*.3.1, *F*.3.2 in Hypothesis 2.5.

2 Recall conditions *F*.3.1, *F*.3.2 in Hypothesis 2.5.

## References

[1] Bambusi D (2003). Birkhoff normal form for some nonlinear PDEs. Commun. Math. Phys..

[2] Bambusi D, Craig W (2008). A Birkhoff normal form theorem for some semilinear PDEs. Hamiltonian Dynamical Systems and Applications. NATO Science for Peace and Security Series.

[3] Bambusi D (2013). Asymptotic stability of ground states in some Hamiltonian PDEs with symmetry. Commun. Math. Phys..

[4] Bambusi D, Delort J-M, Grébert B, Szeftel J (2007). Almost global existence for Hamiltonian semilinear Klein–Gordon equations with small Cauchy data on Zoll manifolds. Commun. Pure Appl. Math..

[5] Bambusi D, Grébert B (2006). Birkhoff normal form for partial differential equations with tame modulus. Duke Math. J..

[6] Bambusi D, Grébert B (2003). Forme normale pour NLS en dimension quelconque. C.R. Math..

[7] Bambusi D, Langella B, Montalto R (2022). Spectral asymptotics of all the eigenvalues of Schrödinger operators on flat tori. Nonlinear Anal. Theory Methods Appl..

[8] Bambusi D, Langella B, Montalto R (2022). Growth of Sobolev norms for unbounded perturbations of the Schrödinger equation on flat tori. J. Differ. Equ..

[9] Bambusi, D., Langella, B.: Growth of Sobolev norms in quasi integrable quantum systems. Preprint arXiv:2202.04505 (2022)

[10] Bernier J, Faou E, Grébert B (2020). Long time behavior of the solutions of NLW on the $$d$$-dimensional torus. Forum Math. Sigma.

[11] Bernier J, Feola R, Grébert B, Iandoli F (2021). Long-time existence for semi-linear beam equations on irrational tori. J. Dyn. Differ. Equ..

[12] Berti M, Delort JM (2018). Almost Global Existence of Solutions for Capillarity-Gravity Water Waves Equations with Periodic Spatial Boundary Conditions.

[13] Berti M, Feola R, Franzoi L (2021). Quadratic life span of periodic gravity-capillary water waves. Water Waves.

[14] Berti M, Feola R, Pusateri F (2022). Birkhoff normal form and long time existence for periodic gravity water waves. Commun. Pure Appl. Math..

[15] Berti M, Maspero A (2019). Long time dynamics of Schrödinger and wave equations on flat tori. J. Differ. Equ..

[16] Biasco L, Massetti JE, Procesi M (2019). Exponential and sub-exponential stability times for the NLS on the circle. Atti Accad. Naz. Lincei, Cl. Sci. Fis. Mat. Nat..

[17] Biasco L, Massetti JE, Procesi M (2020). An abstract Birkhoff normal form theorem and exponential type stability of the 1d NLS. Commun. Math. Phys..

[18] Bourgain J (1999). Global Solutions of Nonlinear Schrödinger Equations.

[19] Bourgain J (1996). Construction of approximative and almost periodic solutions of perturbed linear Schrödinger and wave equations. Geom. Funct. Anal..

[20] Bourgain J (1999). On growth of Sobolev norms in linear Schrödinger equations with smooth time dependent potential. J. Anal. Math..

[21] Colliander J, Keel M, Staffilani G, Takaoka H, Tao T (2010). Transfer of energy to high frequencies in the cubic defocusing nonlinear Schrödinger equation. Invent. Math..

[22] Cong H, Mi L, Wang P (2020). A Nekhoroshev type theorem for the derivative nonlinear Schrödinger equation. J. Differ. Equ..

[23] Delort JM (2009). On long time existence for small solutions of semi-linear Klein–Gordon equations on the torus. J. Anal. Math..

[24] Delort, J.M.: A quasi-linear Birkhoff Normal Forms method. Application to the quasi-linear Klein-Gordon equation on $$\mathbb{S} ^1$$. Astérisque **341** (2012)

[25] Delort JM (2010). Growth of Sobolev norms of solutions of Linear Schrödinger equations on some compact manifolds. Int. Math. Res. Not..

[26] Delort, J.-M.: Quasi-linear perturbations of Hamiltonian Klein–Gordon equations on spheres, volume 234(1103). Memoirs of the American Mathematical Society (2015)

[27] Delort JM, Imekraz R (2017). Long-time existence for the semilinear Klein–Gordon equation on a compact boundary-less Riemannian manifold. Commun. Partial Differ. Equ..

[28] Delort J-M, Szeftel J (2004). Long-time existence for small data nonlinear Klein–Gordon equations on tori and spheres. Int. Math. Res. Not..

[29] Faou E, Gauckler L, Lubich C (2013). Sobolev stability of plane wave solutions to the cubic nonlinear Schrödinger equation on a torus. Commun. Partial Differ. Equ..

[30] Faou E, Grébert B (2013). A Nekhoroshev-type theorem for the Nonlinear Schrödinger equation on the torus. Anal. PDE.

[31] Feola R, Grébert B, Iandoli F (2023). Long time solutions for quasilinear Hamiltonian perturbations of Schrödinger and Klein–Gordon equations on tori. Anal. PDE.

[32] Feola R, Iandoli F (2021). Long time existence for fully nonlinear NLS with small Cauchy data on the circle. Ann. Sc. Norm. Super. Pisa Cl. Sci..

[33] Feola R, Iandoli F, Murgante F (2022). Long-time stability of the quantum hydrodynamic system on irrational tori. Math. Eng..

[34] Feola R, Massetti JE (2023). Sub-exponential stability for the beam equation. J. Differ. Equ..

[35] Feola R, Montalto R (2022). Quadratic lifespan and growth of Sobolev norms for derivative Schrödinger equations on generic tori. J. Differ. Equ..

[36] Giuliani, F.: Sobolev instability in the cubic NLS equation with convolution potentials on irrational tori. Preprint arXiv:2308.13468 (2023)

[37] Giuliani F, Guardia M (2022). Sobolev norms explosion for the cubic NLS on irrational tori. Nonlinear Anal..

[38] Guardia M, Haus E, Hani Z, Maspero A, Procesi M (2022). Strong nonlinear instability and growth of Sobolev norms near quasiperiodic finite-gap tori for the 2D cubic NLS equation. J. Eur. Math. Soc..

[39] Guardia M, Kaloshin V (2015). Growth of Sobolev norms in the cubic defocusing nonlinear Schrödinger equation. J. Eur. Math. Soc. (JEMS).

[40] Hani Z (2014). Long-time instability and unbounded Sobolev orbits for some periodic nonlinear Schrödinger equations. Arch. Ration. Mech. Anal..

[41] Hrabski, A., Pan, Y., Staffilani, G., Wilson, B.: Energy transfer for solutions to the nonlinear Schrödinger equation on irrational tori. Preprint arXiv:2107.01459 (2021)

[42] Ifrim M, Tataru D (2017). The lifespan of small data solutions in two dimensional capillary water waves. Arch. Ration. Mech. Anal..

[43] Ionescu AD, Pusateri F (2019). Long-time existence for multi-dimensional periodic water waves. Geom. Funct. Anal..

[44] Planchon F, Tzvetkov N, Visciglia N (2017). On the growth of Sobolev norms for NLS on $$2$$- and $$3$$-dimensional manifolds. Anal. PDE.

[45] Sohinger V (2011). Bounds on the growth of high Sobolev norms of solutions to nonlinear Schrödinger equations on $${\mathbb{R} }$$. Indiana Univ. Math. J..

[46] Staffilani G, Wilson B (2020). Stability of the cubic nonlinear Schrodinger equation on an irrational torus. SIAM J. Math. Anal..

[47] Yuan X, Zhang J (2014). Long time stability of Hamiltonian partial differential equations. SIAM J. Math. Anal..

